# Loss of *ATRX* promotes aggressive features of osteosarcoma with increased NF-**κ**B signaling and integrin binding

**DOI:** 10.1172/jci.insight.151583

**Published:** 2022-09-08

**Authors:** Suzanne Bartholf DeWitt, Sarah Hoskinson Plumlee, Hailey E. Brighton, Dharshan Sivaraj, E.J. Martz, Maryam Zand, Vardhman Kumar, Maya U. Sheth, Warren Floyd, Jacob V. Spruance, Nathan Hawkey, Shyni Varghese, Jianhua Ruan, David G. Kirsch, Jason A. Somarelli, Ben Alman, William C. Eward

**Affiliations:** 1 Department of Orthopaedic Surgery and; 2 Department of Medicine, Duke University Medical Center, Durham, North Carolina, USA.; 3 Computer Science Department, The University of Texas at San Antonio, San Antonio, Texas, USA.; 4 Department of Biomedical Engineering, Duke University, Durham, North Carolina, USA.; 5 Duke Cancer Institute, Duke University Medical Center, Durham, North Carolina, USA.; 6 Department of Mechanical Engineering and Materials Science, Duke University, Durham, North Carolina, USA.; 7 Department of Pharmacology and Cancer Biology and; 8 Department of Radiation Oncology, Duke University Medical Center, Durham, North Carolina, USA.; 9 College of Veterinary Medicine, North Carolina State University, Raleigh, North Carolina, USA.

**Keywords:** Oncology, Cell migration/adhesion, Extracellular matrix, Integrins

## Abstract

Osteosarcoma (OS) is a lethal disease with few known targeted therapies. Here, we show that decreased *ATRX* expression is associated with more aggressive tumor cell phenotypes, including increased growth, migration, invasion, and metastasis. These phenotypic changes correspond with activation of NF-κB signaling, extracellular matrix remodeling, increased integrin αvβ3 expression, and ETS family transcription factor binding. Here, we characterize these changes in vitro, in vivo, and in a data set of human OS patients. This increased aggression substantially sensitizes *ATRX*-deficient OS cells to integrin signaling inhibition. Thus, *ATRX* plays an important tumor-suppression role in OS, and loss of function of this gene may underlie new therapeutic vulnerabilities. The relationship between *ATRX* expression and integrin binding, NF-κB activation, and ETS family transcription factor binding has not been described in previous studies and may impact the pathophysiology of other diseases with *ATRX* loss, including other cancers and the ATR-X α thalassemia intellectual disability syndrome.

## Graphical abstract

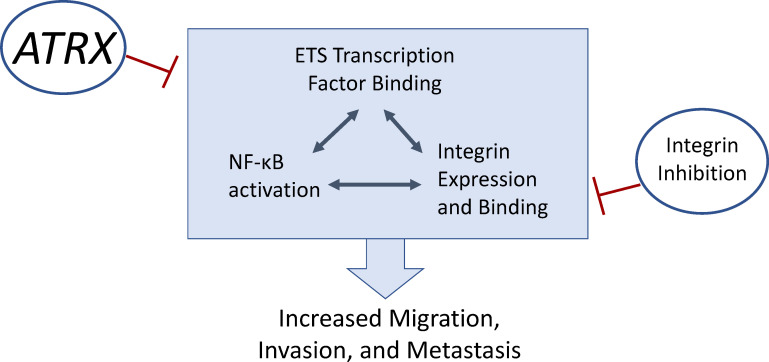

## Introduction

Osteosarcoma (OS) is the most common primary bone cancer diagnosed in humans and is most common among children and adolescents. It is a highly lethal cancer with a propensity for lung metastasis. At present, at least one-third of people diagnosed with OS die from this disease, even if a diagnosis is made and aggressive treatment with surgical excision and combination chemotherapy (i.e., doxorubicin, methotrexate, and cisplatin) is started early in the course of the disease (1, 2). Even for patients who do respond to therapy and survive OS, a normal life expectancy is unlikely, due to the toxicity of current treatment regimens. For patients who develop metastatic disease, the prognosis is particularly bleak: almost 70% of patients do not survive beyond 5 years (3). Despite advances in understanding the molecular and genetic features underlying OS, patient outcomes have not improved significantly in over 30 years. Progress in OS research has been hindered, in part, by the rarity of the disease, with fewer than 1000 human cases diagnosed annually in the United States (2), as well as by the genomic complexity of OS, with its considerable inter- and intratumoral heterogeneity (4–7). For all of these reasons, there remains an urgent need for a better understanding of the molecular underpinnings of OS biology and the resulting altered cellular pathways that may be targetable to provide more specific, refined, and effective therapies for OS.

**Figure 1:**
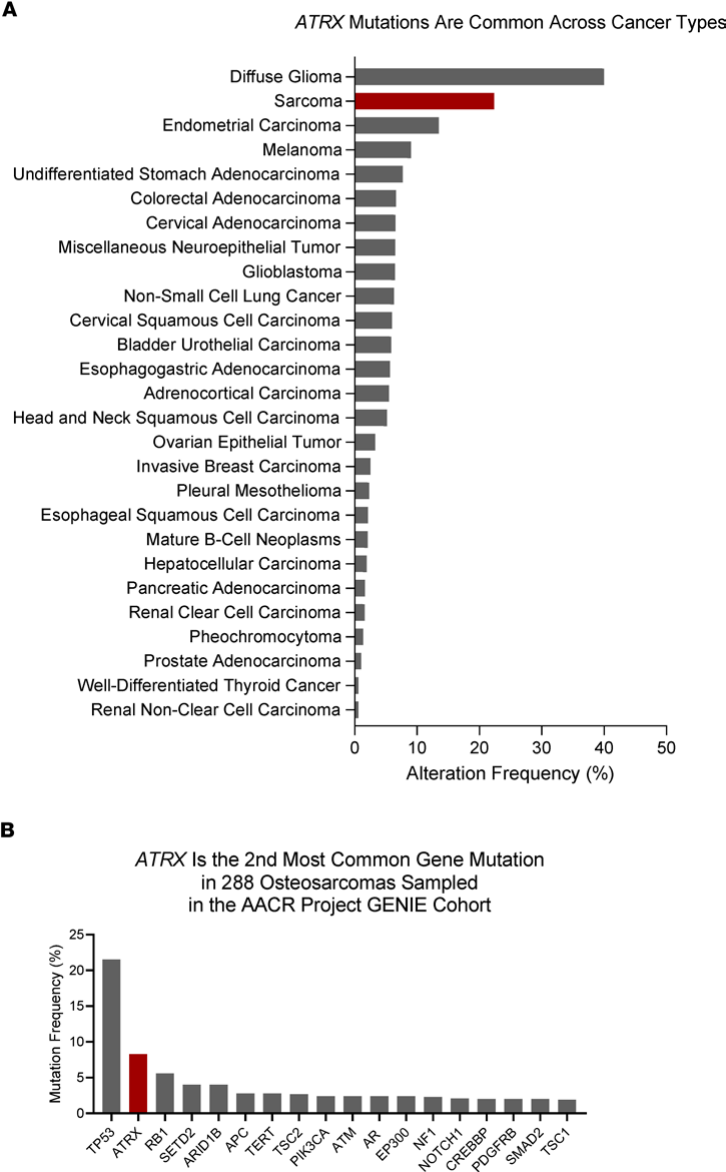
Frequency of *ATRX* mutations. (**A**) Frequency of *ATRX* mutations across cancers in TCGA Pan-Cancer Atlas, as accessed from cBioPortal (17–19). (**B**) *ATRX* is the second most frequently mutated gene in 288 osteosarcomas surveyed by the AACR Project GENIE Consortium as accessed from cBioPortal (18–20).

Recent genomics studies have revealed a high rate of structural variation among OS tumors, including somatic mutations and copy number alterations, as well as a variety of single nucleotide variations or recurrent point mutations (5, 6, 8). Although the *TP53* and *RB1* genes show the most common recurrent alterations in OS, there are also commonly recurrent somatic alterations in other candidate driver genes, such as *ATRX* (5, 9). In fact, across a variety of cancers, several recent studies have identified frequent loss-of-function mutations in *ATRX*, including in gliomas, pancreatic neuroendocrine tumors, melanomas, and soft tissue sarcomas (10–16) (Figure 1A). The Cancer Genome Atlas identified *ATRX* as the 14th most frequently altered gene across all cancers surveyed in the study (17–19). In 288 OS tumors surveyed by the American Association for Cancer Research Project Genomics Evidence Neoplasia Information Exchange (GENIE) Consortium, *ATRX* was one of the most frequently mutated genes, second only to *TP53* (18–20) (Figure 1B). Additionally, at the protein level, several recent studies examining both human and canine OS tumors have found that between 20% and 30% lack nuclear expression of ATRX (11, 21, 22). Importantly, the actual incidence of *ATRX* mutation may be underestimated by modern next-generation sequencing technologies because these methods do not detect many complex indels with short sequence reads. Indeed, Ye et al. (23) identified *ATRX* as one of several oncogenic driver genes with frequent somatic complex indels in tumors across several cancer types that were overlooked by studies using next-generation sequencing modalities. Despite the known frequency and consistency across both human and canine OS, the impact of this *ATRX* loss on OS biology is not fully understood.

α Thalassemia and intellectual disability syndrome X-linked (*ATRX*) is a member of the SWI/SNF family of chromatin remodeling factors. In humans, germline loss of function of the *ATRX* gene causes the α thalassemia and intellectual disability, X-linked syndrome, for which the gene is named (24). The ATRX protein contains 2 highly conserved domains: the SWI/SNF helicase domain, which regulates chromatin remodeling, and the ATRX-DNMT3-DNMT3L (ADD) domain, which controls DNA methylation patterns and transcriptional repression (25, 26). With this ADD domain, ATRX forms dimers with death domain–associated protein (DAXX), and this complex acts as a histone chaperone to deposit histone variant H3.3 to GC-rich regions of the genome, including the pericentric, ribosomal, and telomeric repeat sequences (27, 28). In the context of cancers, nearly all published studies have focused on the correlation between loss of *ATRX* expression and activation of the alternative lengthening of telomeres (ALT) pathway for telomere maintenance (21, 22, 29, 30). However, while replicative immortality is one of the key hallmarks of cancer (31), it is not likely to be solely sufficient to promote oncogenesis. Very recently, a few studies have explored other tumor-promoting changes that occur in cancers with *ATRX* deficiency, including increased cellular motility in glioma cells and TGF-β activation with *CDH1* (E-cadherin) downregulation in liver cancer cells (16, 32, 33).

Based on its frequent loss in OS and given the important role of *ATRX* in chromatin remodeling and methylation patterns, we sought to define the phenotypic and mechanistic impacts of *ATRX* loss in OS. We hypothesized that loss of *ATRX* would increase aggressive cellular features of OS, including tumor initiation, migration, invasion, and metastasis, and we describe here our investigations into the impact of this *ATRX* loss on OS biology, using a range of models to examine each specific cellular phenotype of aggression. Using RNA-Seq and ATAC-Seq, we examined changes in cellular pathways that correspond with *ATRX* loss. These analyses pinpointed alterations in the NF-κB and several extracellular matrix–related (ECM-related) pathways. Analysis of chromatin binding motif enrichments identified common overlap of ATRX binding sites with ETS family transcription factor motifs, which is notable because ETS family proteins play an important role in osteogenic differentiation (34, 35). Using high-throughput collateral sensitivity screens, we found that OS cells with *ATRX* KO were sensitized to an integrin inhibitor. Further examination of these cells demonstrated increased integrin β3 expression with *ATRX* loss. Treatment of *ATRX*-KO cells with the integrin inhibitor was sufficient to reverse the phenotypes of aggression, particularly migration and invasion, and this drug partially reversed the nuclear upregulation of the NF-κB transcription factors. Examining publicly available pancancer sequencing data, we similarly found enriched integrin signaling with *ATRX* alteration. The relationship between *ATRX* expression and integrin binding, NF-κB activation, and ETS family transcription factor binding may impact other known diseases with *ATRX* loss, including other cancers. Our data show that *ATRX* mutations sensitize OS cells to integrin inhibition. Future studies are needed to explore integrin inhibition as a potentially new targeted therapy for *ATRX*-deficient OS.

## Results

### ATRX loss promotes tumor initiation.

**Figure 2:**
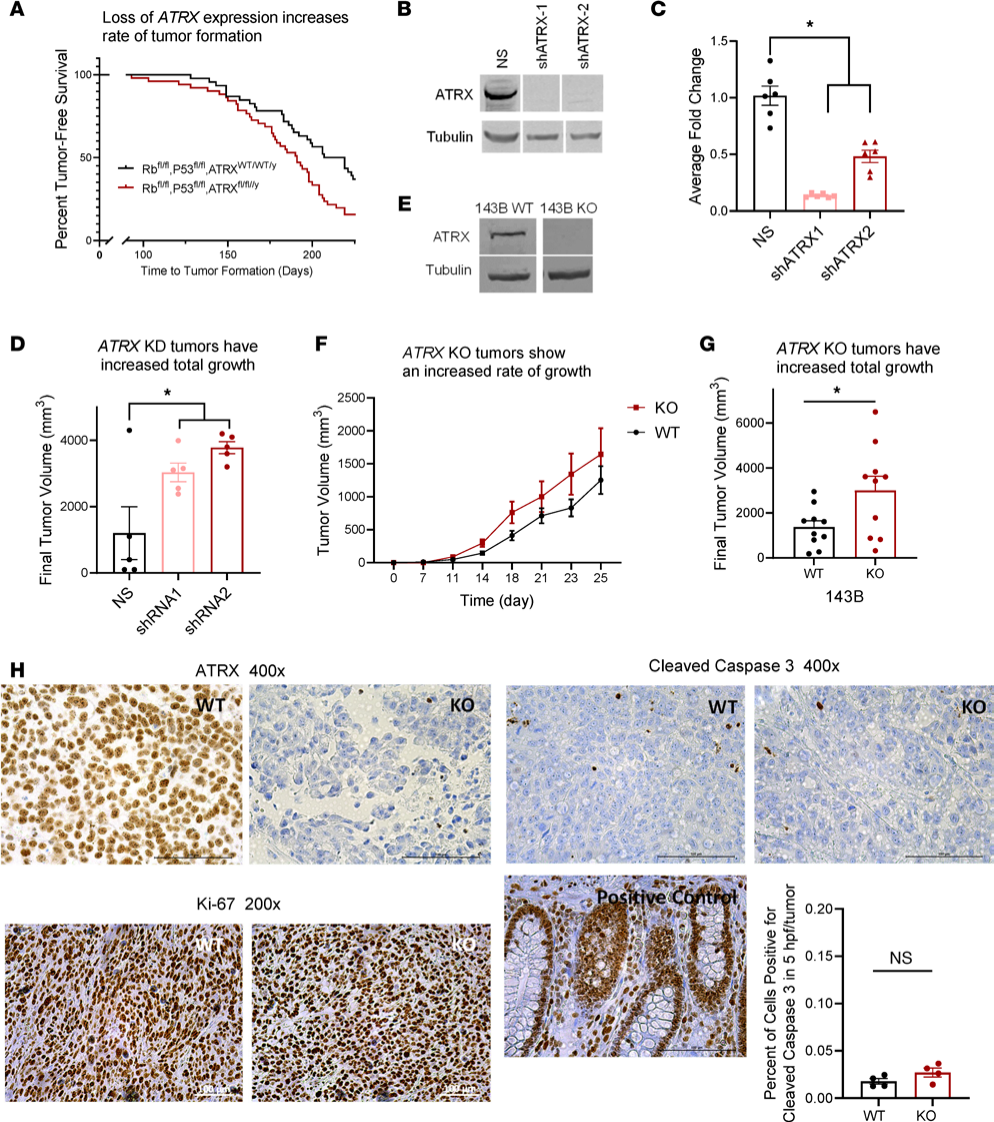
*ATRX* loss promotes tumor initiation and growth. (**A**) Loss of *ATRX* expression increased the rate of tumor formation in an *Osterix-Cre*–driven mouse model of OS. Kaplan-Meier, log-rank *P* = 0.0021, experimental cohorts *Osx-Cre^+^p53^fl/fl^Rb^fl/fl^* (*n* = 26 males and 25 females) or *Osx-Cre^+^p53^fl/fl^Rb^fl/fl^ATRX^fl/fl/y^* (*n* = 22 males and 25 females). (**B** and **C**) Western blot and qPCR results showing knockdown (KD) of *ATRX* with 2 shRNA constructs in the 143B human OS cell line. Dunnett’s multiple-comparison test, *P* < 0.0001 for both NS versus shATRX-1 and NS versus shATRX-2 for qPCR results. (**D**) Established xenografts of the 143B human OS cell line with *ATRX* shRNA KD showed greater final tumor volume compared with NS controls (1-way ANOVA with multiple comparisons, *P* = 0.04 shATRX-1 versus NS, *P* = 0.006 shATRX-2 versus NS; *n* = 3 males and 2 females per treatment group). (**E**) Western blot results show effective CRISPR-Cas9 KO of *ATRX* expression in the 143B human OS cell line. (**F** and **G**) Established xenografts of the 143B cell line with *ATRX* KO had a faster rate of tumor growth (1-way repeated-measures ANOVA, log transform, *P* = 0.02) and larger final tumor volumes compared with WT cells (Student’s *t* test, *P* = 0.03; *n* = 5 males and 5 females per treatment group). (**H**) Histology of xenograft tumors. The expected *ATRX* expression status of all WT and KO tumors was validated by IHC for *ATRX*. All xenograft tumors stained strongly for Ki-67 (greater than 95% positive), suggesting high cellular proliferation in all tumors. No significant differences were observed in the xenograft tumors with IHC staining for cleaved caspase 3 with less than 5% of cells staining positively in all tumors. Positive control is shown.

We hypothesized that *ATRX* loss of expression in OS would correlate with the acquisition of aggressive tumor phenotypes, including alterations in tumor initiation, growth, migration and invasion, and metastasis (Supplemental Figure 2A; supplemental material available online with this article; https://doi.org/10.1172/jci.insight.151583DS1). We used a range of in vivo and in vitro models to examine each of these specific phenotypes. To examine how *ATRX* loss alters tumor initiation in OS, we chose to work with a previously established transgenic *Osterix*-*Cre* mouse model with conditional (floxed) alleles of both *p53* (*p53^fl/fl^*) and *Rb* (*Rb^fl/fl^*) and with a Tet-off cassette providing an additional level of temporal control (36, 37). The transgene expression of Cre recombinase is driven by promoter sequences of *Osterix*, a principal regulator of bone differentiation, and is therefore mostly restricted to committed osteoblast progenitors (36, 38) (although recent studies show *Osterix* expression in additional subsets of cells; refs. 39, 40). These mice with homozygous deletion of *Rb* and *p53* show completely penetrant OS development, typically between 4 and 8 months of age (37), occurring most frequently in the jaw and head, rear limb, hip, ribs, and vertebra. To determine if *Atrx* loss would decrease the time to tumor initiation, we added a floxed *Atrx* allele (*Atrx^fl/fl/y^*) to the *Osx-Cre^+^p53^fl/fl^Rb^fl/fl^* to create *Osx-Cre^+^p53^fl/fl^Rb^fl/fl^Atrx^fl/fl/y^*. We removed a doxycycline diet at time of weaning and monitored mice of each genotype for tumor development. To more comprehensively identify tumors that developed in any bone, we performed monthly fluoroscopy on a subcohort of 10 females and 10 males of each genotype to further increase our sensitivity in tumor detection (Supplemental Figure 1A). Tumors were collected, and gene recombination of tumors was confirmed by PCR to distinguish the KO allele from the floxed, nonrecombined allele (Supplemental Figure 1B). Consistent with our hypothesis, loss of *Atrx* significantly increased the rate of tumor initiation compared with the *p53*/*Rb* KO alone (Figure 2A; log-rank, *P* = 0.0021).

### ATRX loss promotes tumor growth.

Given the role of *ATRX* loss in speeding the time to tumor initiation, we next sought to determine if *ATRX* loss would lead to increased tumor growth. To do this, we chose to compare tumor growth using a xenograft mouse model in which tumors would be easily detected and would develop in the same location (in the s.c. flank) for all mice. We stably transduced human 143B OS cells with a nonsilencing (GFP) shRNA or 1 of 2 independent shRNA constructs targeting *ATRX* (shATRX-1 and shATRX-2). *ATRX* knockdowns were confirmed via Western blotting and quantitative PCR (qPCR) (Figure 2, B and C). We then injected the control and *ATRX* shRNA knockdown 143B cells s.c. in SCID-beige mice and monitored tumor growth rates over time. *ATRX* knockdown significantly enhanced tumor growth in these xenografts for both shRNAs (Figure 2D). To further validate these findings, we also developed a CRISPR-Cas9 KO of *ATRX* in the 143B human OS cell line (Figure 2E and Supplemental Figure 2B), and we repeated the previous xenograft experiment with *ATRX*-KO or WT cells injected s.c. in SCID-beige mice. Consistent with the results of the shRNA-mediated knockdown study, *ATRX* KO increased both the rate of tumor growth and final tumor volume as compared with WT 143B cells (Figure 2, F and G).

### Histological analysis of xenograft tumors and in vitro cells show no significant differences in cell proliferation or apoptosis with ATRX loss.

In order to investigate whether the increased tumor size found with *ATRX* KO was due to differences in tumor cell proliferation or apoptosis, IHC was performed on the formalin-fixed, paraffin-embedded xenograft tumors harvested from these mice. First, we did verify by IHC that the *ATRX* KO was retained in the final tumors harvested (Figure 2H). Both WT and KO tumors stained very strongly for Ki-67, with greater than 95% of cells staining positive, supporting high cellular proliferation in all tumors (Figure 2H). The 143B cell line is known to be an especially proliferative cell line, and perhaps this high baseline of proliferation limits the ability to detect differences in proliferation, if present, between tumors derived from WT and *ATRX*-knockdown cells. Additional in vitro studies of the 143B nonsilenced or shRNA knockdown cells examining change in percent confluence over time in the Incucyte live cell imager showed similarly high proliferation rates of all cells, with no significant differences across cell types (Supplemental Figure 2C). Thus, we were unable to conclude that the tumor size differences were due to changes in rate of proliferation. We then performed IHC for cleaved caspase 3 to compare apoptosis, but again, no significant differences were found, with less than 5% of cells for either KO or WT tumors exhibiting positive staining (Figure 2H). Thus, we were also unable to conclude that the tumor size differences were due to resistance to apoptosis.

### ATRX loss promotes tumor migration and invasion.

**Figure 3:**
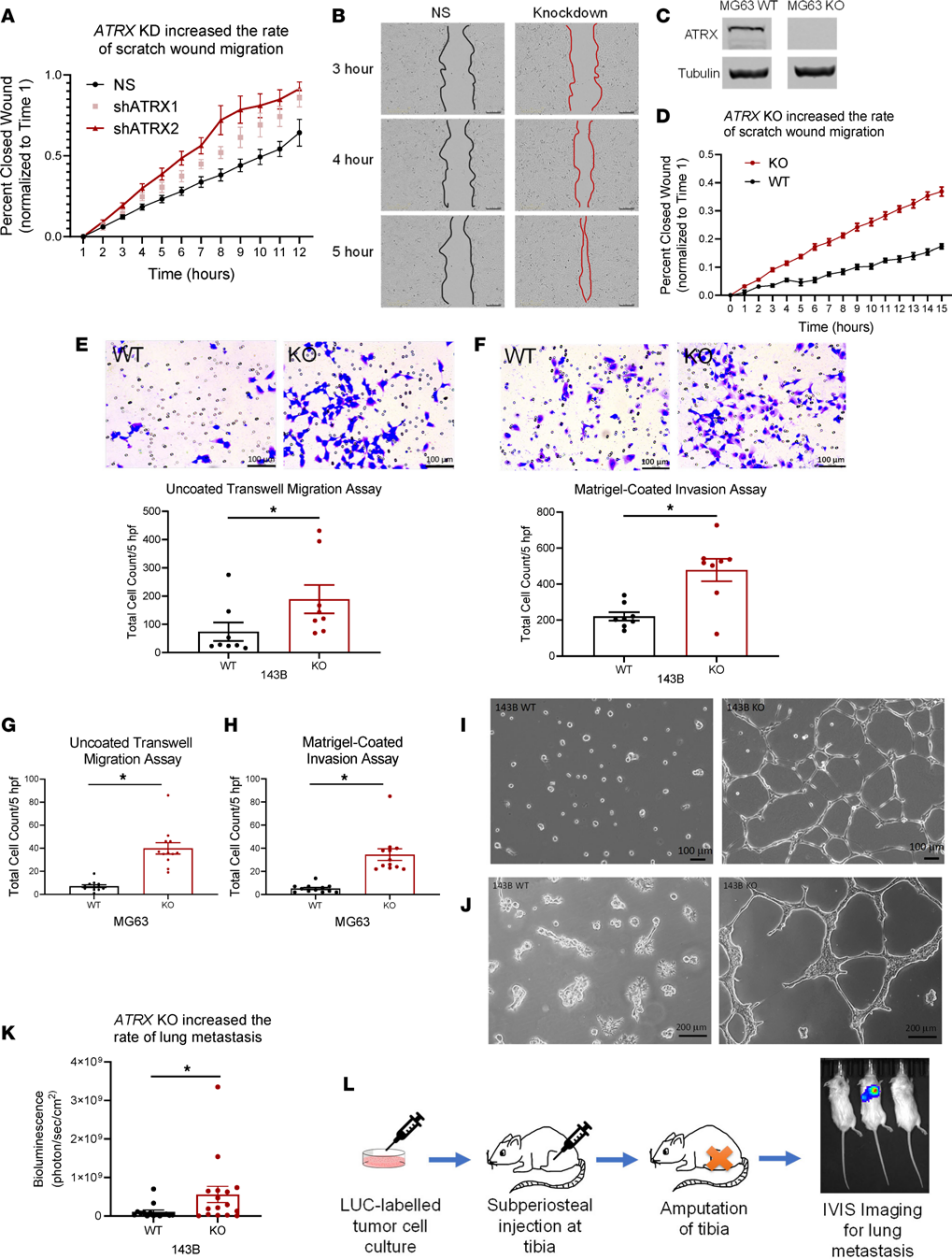
Tumor migration, invasion, and rate of metastasis increase with loss of *ATRX* expression. (**A**) In a scratch wound assay, 143B cells with shRNA KD of *ATRX* showed faster wound closure than the nonsilenced (NS) control cells (1-way repeated-measures ANOVA, *P* < 0.0001 for both shATRX-1 and shATRX-2 compared with NS; *n* = 8 replicates per cell type; 2 experiments). (**B**) Representative images of scratch wounds at 3 time points. Scale bars: 200 μm. (**C**) Western blot showing effective CRISPR-Cas9 KO of *ATRX* expression in the MG-63 human OS cell line. (**D**) Similarly, in the MG-63 cell line, KO cells showed significantly faster wound closure than WT cells (1-way repeated-measures ANOVA, *P* < 0.0001; WT, *n* = 13 replicates; KO, *n* = 10 replicates; 2 experiments). (**E** and **F**) 143B-KO cells showed increased migration and invasion, respectively, in uncoated and Matrigel-coated transwell plates (1-tailed Student’s *t* test [**E**] and 2-tailed Student’s *t* test [**F**]; uncoated: *P* = 0.04, *n* = 8 replicates; Matrigel: *P* = 0.002, *n* = 8 replicates; 2 experiments). Scale bars: 100 μm. (**G** and **H**) MG-63–KO cells also showed increased migration and invasion, respectively, in uncoated and Matrigel-coated transwell assays (2-tailed Student’s *t* test; uncoated: *P* < 0.0001, *n* = 12 replicates; Matrigel: *P* < 0.0001, *n* = 12 replicates; 2 experiments). (**I**) After 24 hours of growth on a bed of Matrigel, WT cells remain in tight clusters, whereas KO cells form a network of connecting “tubes” or branching networks through the matrix. Scale bars: 100 μm. (**J**) After 96 hours of growth, the distinct differences in morphology between the 143B WT and KO cells are even more apparent. Scale bars: 200 μm. (**K**) Luciferase-labeled 143B WT or KO cells were injected into the subperiosteal space of the tibia of SCID-beige mice. *ATRX* KO correlates with an increased rate of lung metastasis at 1 week after amputation (1-tailed Student’s *t* test, *P* = 0.026; WT: *n* = 5 males and 10 females; KO: *n* = 6 males and 11 females). (**L**) Experimental design for orthotopic injections with LUC-labeled cells to study lung metastasis.

We next decided to investigate the impact of *ATRX* loss on changes in cell motility, including migration and invasion, using standard in vitro scratch wound and transwell migration/invasion chamber assays. Assays were repeated with 2 well-established OS cells lines, 143B and MG-63, to test for consistency despite the different genetic mutation background found in each cell line. To examine migration, we first performed scratch wound assays with human 143B cells with shRNA-mediated knockdown of *ATRX* and with human MG-63 cells with CRISPR/Cas9-mediated KO of *ATRX* (Figure 3, A–D, and Supplemental Figure 2B). For both cell lines, wound closure rate was significantly increased with *ATRX* knockdown/KO, supporting increased migration in *ATRX*-null OS cells. We also tested if *ATRX* loss would increase transwell migration/invasion using Boyden chamber migration/invasion assays. As with the scratch wound assays, CRISPR/Cas9-mediated KO of *ATRX* in both the 143B and MG-63 cell lines increased both migration and Matrigel invasion (Figure 3, E–H).

Interestingly, when we plated the 143B WT and KO cells on a bed of Matrigel, there was a distinct difference in appearance after 24 hours, and it was even more apparent after 96 hours. KO cells formed a network of connecting “tubes” in the Matrigel, permitting more cell-to-cell contact, whereas the WT cells grew in more confined clusters within the Matrigel (Figure 3, I and J). These findings support an enhanced ability of KO cells to invade through the Matrigel, perhaps by secreting enzymes and ECM proteins to form this branching network. When repeating this experiment with the less-proliferative MG-63 cell line, the differences in morphology were less distinct but still notable after 72 hours (Supplemental Figures 3, A and B).

### ATRX loss promotes tumor metastasis to lungs.

The in vitro migration and invasion assays suggest that *ATRX* loss may promote metastatic dissemination of OS cells. To further examine the role of *ATRX* deficiency in driving metastasis, we used an orthotopic metastasis model of OS. To do this, luciferase-labeled WT or *ATRX*-KO cells were injected into the subperiosteal space of the tibia in SCID-beige mice (Supplemental Figure 2D). These mice developed tibial OS in approximately 2 weeks, after which the affected legs were amputated, and metastatic progression to the lungs was quantified using luciferase imaging with the In Vivo Imaging System (Caliper Life Sciences Inc., PerkinElmer; see Supplemental Methods), a method previously validated in our lab as an accurate and accessible assessment of in vivo metastatic tumor burden (41). Most of the mice developed lung metastases if given enough time; however, consistent with our in vitro migration and invasion assays, at 1 week after amputation, *ATRX* KO led to significantly more lung metastases as compared with WT cells, supporting the conclusion that *ATRX* loss promotes lung metastasis (Figure 3, K and L).

### ATRX loss promotes NF-κB pathway activation and downregulates ECM proteins.

**Figure 4:**
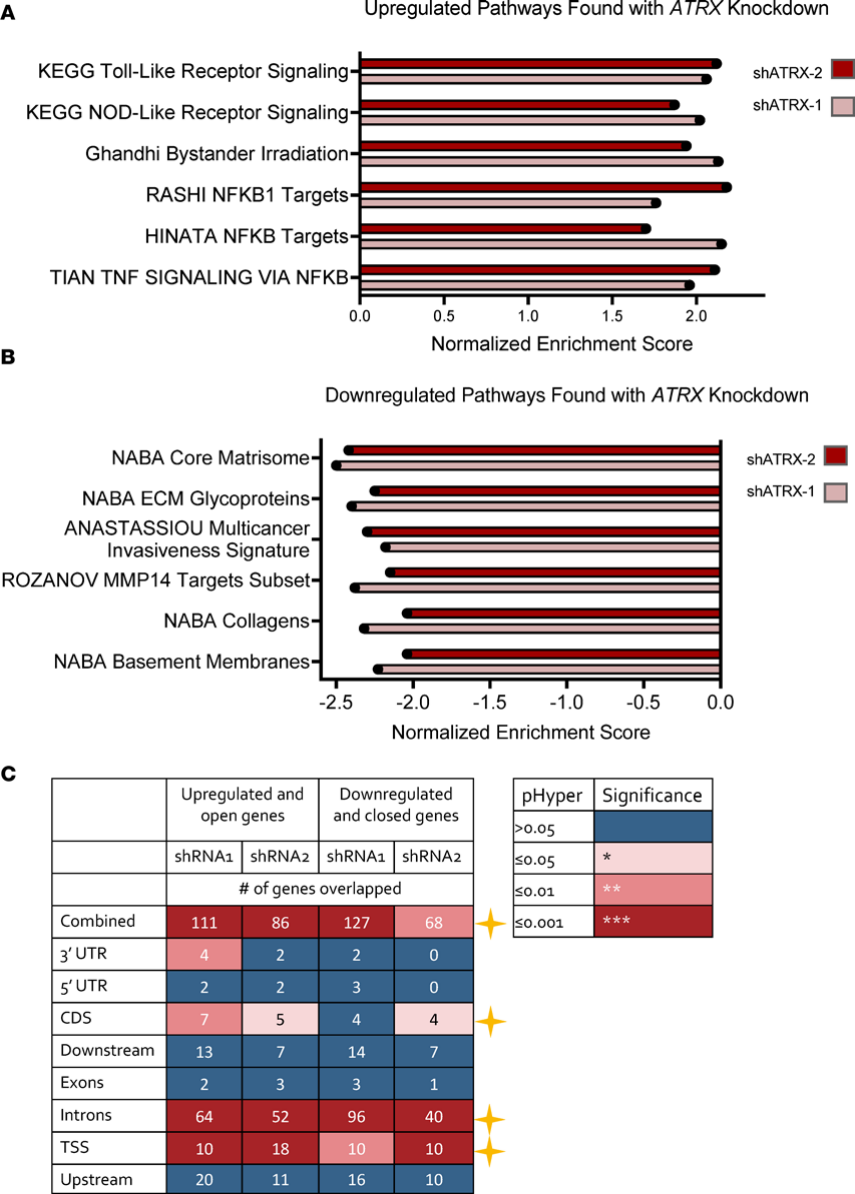
RNA-Seq identifies upregulation of several NF-κB pathways and downregulation of several extracellular matrix (ECM) pathways. (**A** and **B**) RNA-Seq of 143B human OS cells with either the NS control or 1 of 2 shRNA knockdowns of *ATRX* show upregulation of NF-κB pathways and downregulation of various ECM-related pathways with KD of *ATRX* expression. (**C**) ATAC-Seq changes in chromatin openness with *ATRX* shRNA KD correlate with RNA-Seq findings of gene expression changes, particularly when the chromatin peaks are found in regions of introns or transcriptional start sites (Benjamini and Hochberg correction for *P* value calculations as shown). These data suggest that the significant alterations in NF-κB and ECM pathways may derive from *ATRX*-mediated effects on the chromatin state. Gold crosses indicate types of DNA regions that showed the most significant correlations with gene expression changes.

**Figure 5:**
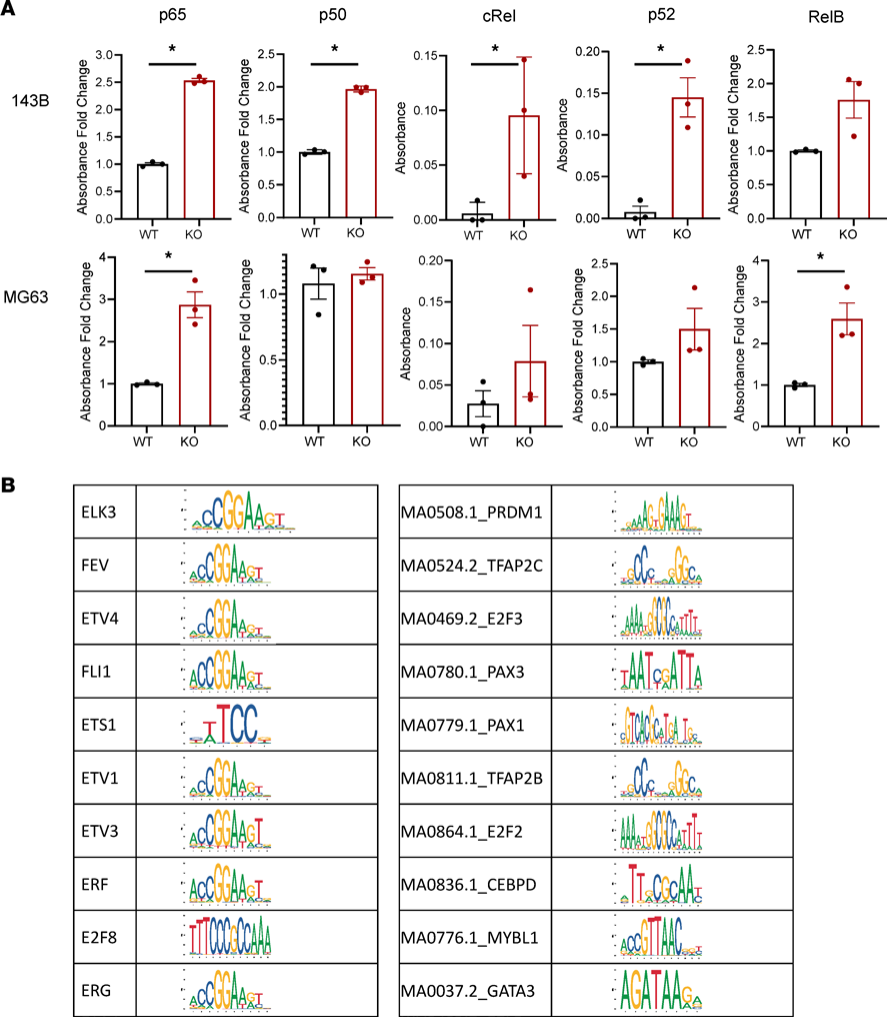
*ATRX* KO increases nuclear expression of NF-κB transcription factors, and an analysis of ATRX chromatin binding motifs highlights common ETS transcription factor family binding motifs. (**A**) NF-κB ELISA shows increased relative nuclear expression of this family of transcription factors (TFs) with *ATRX* KO when comparing nuclear extracts from both the 143B and MG-63 WT or KO cell lines (Multiple *t* tests). Statistically significant comparisons for 143B: p65, *P* = 0.0004; p50, *P* = 0.000009; cRel, *P* = 0.04; p52, *P* = 0.009; *n* = 3 replicates for each cell type and TF. Statistically significant comparisons for MG-63: p65, *P* = 0.01; RelB, *P* = 0.024; *n* = 3 replicates for each cell type and TF. (**B**) Top differentially deviated binding motifs found with *ATRX* shRNA knockdown in the 143B cell line most closely resemble the ETS transcription factor family binding motifs.

Given the important roles of *ATRX* as both a chromatin remodeler and regulator of histone and DNA methylation, we hypothesized that loss of *ATRX* would have broad impacts on gene expression across the genome and that the resulting alterations to multiple cellular pathways would collectively promote more aggressive cancer phenotypes. To examine this, we performed an integrated genomics analysis of RNA-Seq and ATAC-Seq profiles using these nonsilenced or shRNA-knockdown 143B cells. Analysis of the RNA-Seq data by Gene Set Enrichment Analysis pinpointed enrichment of several pathways relevant to OS upon *ATRX* knockdown, including upregulation of the NF-κB pathway and downregulation of ECM proteins (Figure 4, A and B, and Supplemental Figure 4, A and B). To further understand the mechanisms underlying these gene expression alterations, we analyzed genomic changes in chromatin openness using ATAC-Seq. Analysis of the ATAC-Seq findings revealed altered chromatin openness across the genome upon *ATRX* shRNA knockdown compared with the nonsilenced control, supporting the global genomic importance of the role of ATRX as a chromatin remodeler (Supplemental Figure 5A). We then cross-referenced our ATAC-Seq results with our RNA-Seq data and found that transcriptionally upregulated genes were significantly enriched in regions of more open chromatin upon *ATRX* loss (Figure 4C). Similarly, genes that were downregulated with *ATRX* knockdown significantly corresponded with regions of more closed chromatin (Figure 4C). These data suggest that the significant alterations in NF-κB and ECM pathways may derive from *ATRX*-mediated effects on chromatin state. The upregulation of the NF-κB pathway was validated using an ELISA for nuclear extracts of our cell types. Consistent with the RNA-Seq data, NF-κB transcription factor family activity was upregulated in nuclear extracts from both 143B and MG-63 *ATRX*-KO cell lines compared with WT cells (Figure 5A).

### Analysis of common ATRX binding motifs pinpoints ETS family transcription factor binding.

To examine potential common binding motifs for *ATRX* across the genome, we analyzed our sequencing data using chromVar to look at known transcription factor binding motifs in the Jasper motif database as well as all 6 k-mers. The top differentially enriched motifs correspond most closely to ETS family transcription factors, suggesting an important interaction between *ATRX* and these transcription factors (Figure 5B).

### OS cells with ATRX loss display collateral sensitivity to pharmacological inhibition of integrin signaling.

**Figure 6:**
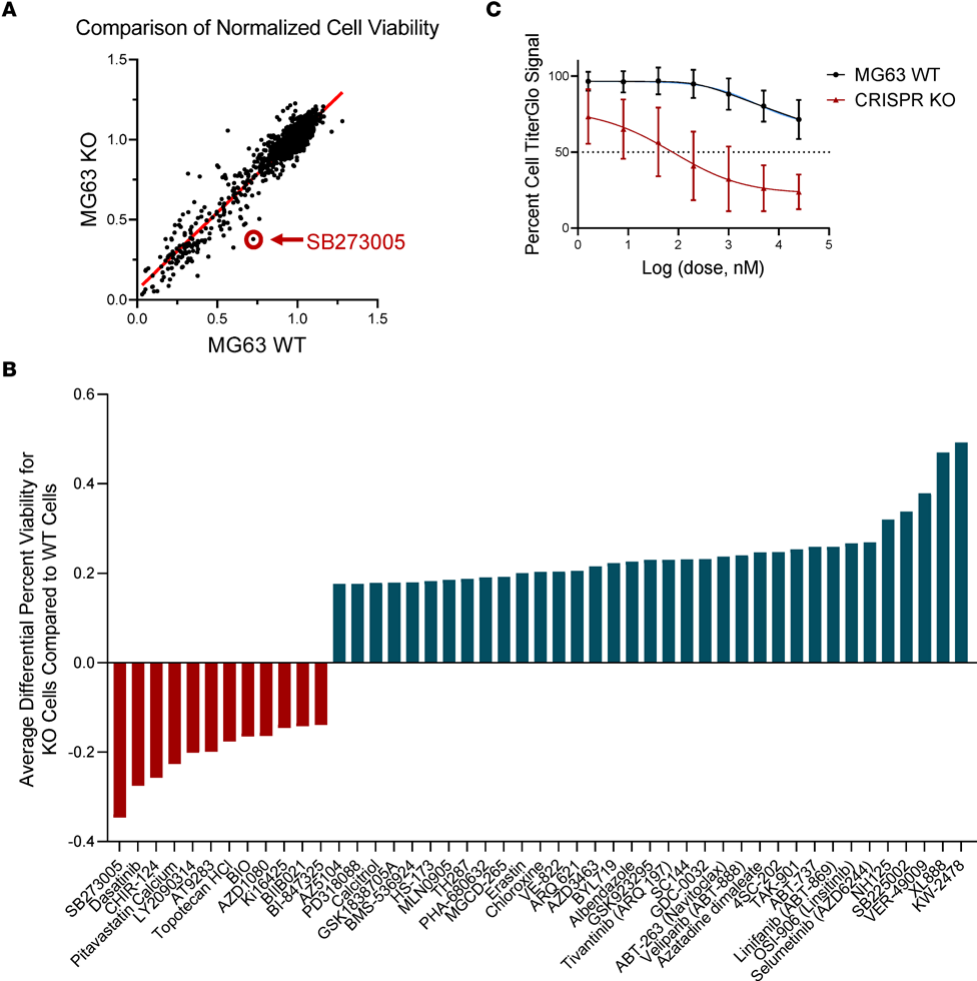
*ATRX* KO sensitizes cells to pharmacological inhibition of integrin signaling in vitro. (**A**) Comparison of normalized cell viability for all 2100 drugs in a bioactive compound screen. *ATRX*-KO cells were most substantially sensitized to the integrin inhibitor SB273005. (**B**) Among the compounds for which this differential viability was outside of the 99th percentile confidence interval, most (74%) displayed increased resistance upon *ATRX* loss. (**C**) SB273005 IC^50^ curves show significant sensitization of KO cells to the integrin inhibitor SB273005. Nonlinear regression, extra sum-of-squares F test, *P* = 0.006.

Our collective data suggest that *ATRX* loss provides a selective advantage to OS cells by reducing barriers to tumor initiation, increasing tumor growth rate, increasing migratory/invasive capacity, and enhancing survival in the metastatic niche. Given the host of adaptations and survival benefits conferred on cells with *ATRX* loss, we hypothesized that these cells would be sensitized to loss of a secondary pathway. To identify potential actionable collateral sensitivities, we performed a high-throughput drug screen of 2100 bioactive compounds on MG-63 WT or KO OS cells. Among the compounds for which this differential viability was outside of the 99th percentile CI, most (74%) displayed increased resistance upon *ATRX* loss (Figure 6, A and B). These compounds included heat shock protein inhibitors KW-2478, XL888, and VER-49009. Interestingly, however, we also observed increased sensitivity of *ATRX*-null cells to the integrin inhibitor SB273005 (Figure 6A). SB273005 is a nonpeptide antagonist of the αvβ3 and αvβ5 integrins (42, 43). We further validated this integrin inhibitor sensitization upon *ATRX* loss using IC_50_ assays, confirming a significant drug sensitization with *ATRX* loss (Figure 6C). Integrins are known key interactors with ECM components and directly activate the NF-κB pathway, all of which is consistent with our observations that both NF-κB and ECM pathways are altered upon *ATRX* loss.

**Figure 7:**
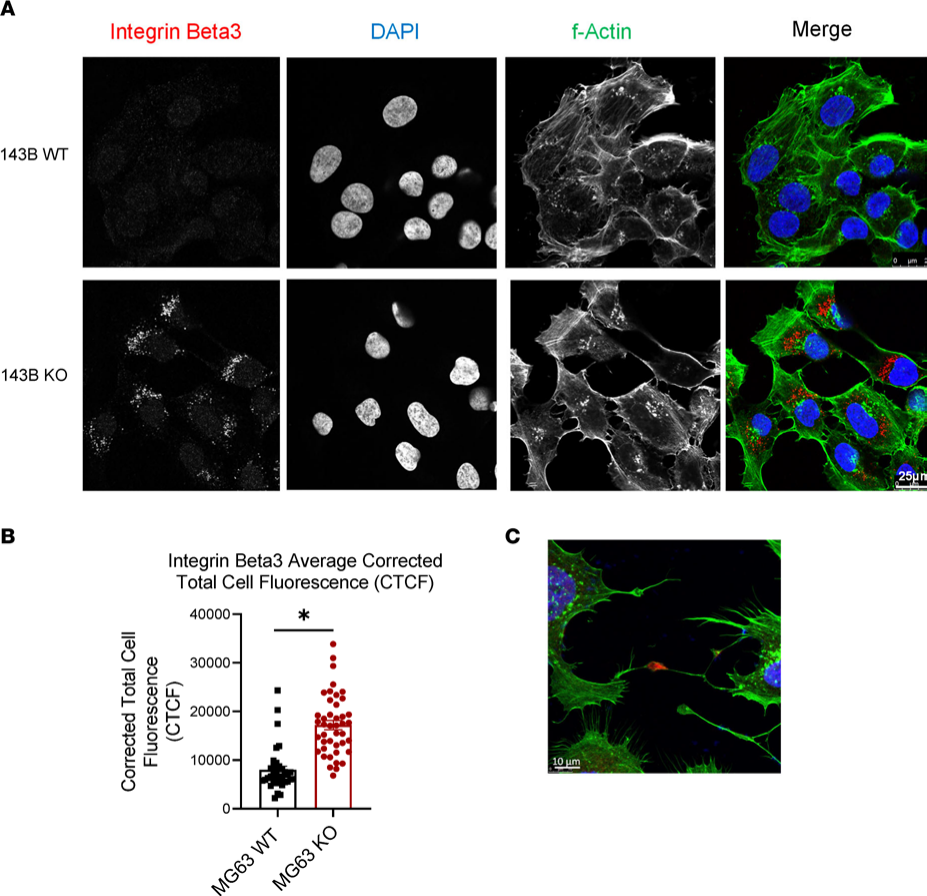
*ATRX*-KO cells have greater expression of integrin β3. (**A** and **B**) 143B *ATRX*-KO cells show significantly increased expression of integrin β3 compared with WT cells (2-tailed Student’s *t* test, *P* < 0.0001; KO, *n* = 49; WT, *n* = 52). Scale bars: 25 μm. (**C**) Example of integrin β3 expression at cell-to-cell adhesions. Scale bar: 10 μm.

The integrin inhibitor SB273005 has a high affinity for αvβ3 integrins; therefore, we hypothesized that there would be an increase in expression of integrin αvβ3 at the cell surface with loss of *ATRX* expression. To test this, we performed immunofluorescent imaging of our cell lines. As predicted, we saw significantly increased expression of integrin β3 in our KO cells in both the 143B and MG-63 cell lines (Figure 7, A–C, and Supplemental Figure 3C). One of the key matrix components to which integrins αvβ3 and αvβ5 bind is osteopontin; therefore, we examined expression of the gene *SPP1*. *SPP1* mRNA expression was upregulated in our *ATRX*-knockdown cells (Supplemental Figure 3D), and we hypothesize that there may be increased secretion of this phosphoprotein correlated with *ATRX* loss. Future studies will further investigate these ECM-integrin relationships and how they correlate with *ATRX* deficiency.

**Figure 8:**
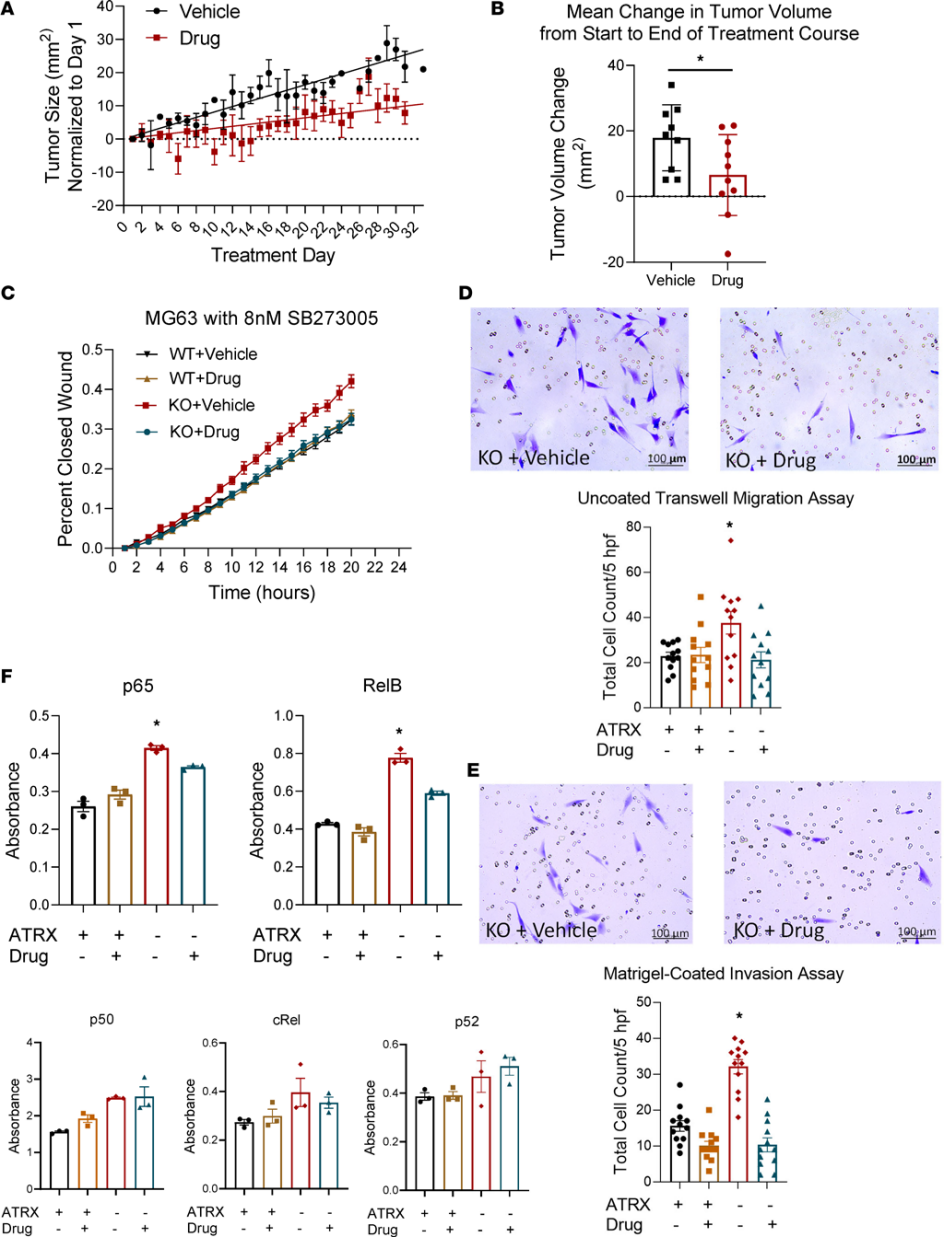
*ATRX* KO sensitizes cells to pharmacological inhibition of integrin signaling in vivo, and integrin inhibition is sufficient to reverse aggressive phenotypes seen with *ATRX* loss. (**A** and **B**) Treatment with the integrin inhibitor SB273005 significantly reduced tumor growth in *ATRX*-null U-2OS cells (1-way repeated-measures ANOVA, *P* < 0.0001 for volume change over time; 1-tailed Student’s *t* test, *P* = 0.02 for final tumor volume change; vehicle-treated: *n* = 4 females, 5 males; integrin inhibitor-treated: *n* = 5 females, 5 males). (**C**) The integrin inhibitor reversed the increased rate of migration conferred by loss of *ATRX* in the MG-63 cell line (repeated-measures ANOVA, *P* < 0.0001; WT vehicle, *n* = 19 replicates; WT drug, *n* = 26 replicates; KO vehicle, *n* = 18 replicates; KO drug, *n* = 25 replicates). (**D** and **E**) The integrin inhibitor SB273005 reversed the increased migration and invasion observed with the MG-63 *ATRX*-KO cells in both uncoated transwell assays (**D**) and Matrigel-coated transwell assays (**E**). Scale bars: 100 μm. (Tukey’s multiple-comparison test for uncoated wells: *ATRX*-KO vehicle versus WT Vehicle *P* = 0.028, KO Vehicle versus WT Drug *P* = 0.035, KO Vehicle versus KO Drug *P* = 0.0117, *n* = 12 wells, 2 experiments. Tukey’s multiple-comparison test for Matrigel-coated wells: *ATRX*-KO vehicle versus WT vehicle, *P* < 0.0001; KO vehicle versus WT drug, *P* < 0.0001; KO vehicle versus KO drug, *P* < 0.0001; *n* = 12 wells; 2 experiments). (**F**) Integrin inhibitor treatment partially reversed the upregulation of NF-κB transcription factors p65 and RelB in the *ATRX*-KO cells (Tukey’s multiple-comparison tests for p65: ATRX-KO vehicle versus WT vehicle, *P* < 0.0001; KO vehicle versus WT drug, *P* < 0.0001; KO vehicle versus KO drug, *P* = 0.028; *n* = 3. Tukey’s multiple-comparison tests for RelB: *ATRX*-KO vehicle versus WT vehicle, *P* < 0.0001; KO vehicle versus WT drug, *P* < 0.0001; KO vehicle versus KO drug, *P* = 0.0002; *n* = 3).

We next tested the integrin inhibitor SB273005 in vivo with xenograft mouse tumors to examine its efficacy as a therapeutic for *ATRX*-deficient OS. For this experiment, we chose to use an established *ATRX*-null OS cell line, U-2 OS, to see if this cell line would respond in vivo as the MG-63–KO cells did in vitro. As observed in our high-throughput in vitro screen that treatment with SB273005 significantly reduced in vivo tumor growth in xenografts formed from s.c. flank injections of these U-2 OS cells (Figure 8, A and B).

### Integrin inhibition is sufficient to reverse aggressive phenotypes seen with ATRX loss.

Given the functional connection between αvβ3 and αvβ5 signaling, NF-κB signaling, and invasive phenotypes in cancer, we tested if integrin signaling inhibition could reverse the increase in phenotypes of aggression that we found with *ATRX* knockdown/KO. We first repeated our scratch wound assays with WT and KO cells treated with vehicle control or with 8 nM SB273005. The KO cells treated with vehicle retained a significant increase in rate of wound closure relative to both vehicle- and drug-treated WT cells; however, the KO cells treated with SB273005 had a wound closure rate that was very similar to the WT cells (Figure 8C and Supplemental Figure 3E). Thus, this experiment supports that the integrin inhibitor is sufficient for reversal of the increased migratory phenotype conferred by loss of *ATRX*. These results suggest that the enhanced migratory capability of *ATRX*-deficient cells is likely due to increased integrin expression and binding. Similarly, treatment of KO cells with the integrin inhibitor reversed the increased migration and invasion observed in the transwell migration and Boyden invasion chamber assays (Figure 8, D and E).

### Integrin inhibition partially reverses upregulation of NF-κB signaling.

Given the reported relationship between integrin binding and NF-κB signaling, we also investigated whether the integrin inhibitor would be sufficient to reverse the upregulation of the NF-κB transcription factor family in the *ATRX*-KO cells. Both KO and WT MG-63 cells were incubated for 24 hours with either SB273005 or the vehicle control, nuclear extractions were performed, and the NF-κB ELISA was repeated. The 2 significantly upregulated transcription factors seen in our prior experiment with the MG-63 KO cells, p65 and RelB, were both significantly rescued by integrin inhibitor treatment compared with vehicle control (Figure 8F). These results support a close correlation between the increased migratory and invasive phenotypes found with *ATRX* deficiency, integrin binding, and NF-κB pathway activation.

### Integrin signaling is enriched in ATRX-altered tumors in ICGC/TCGA Pan-Cancer Analysis of Whole Genomes data set.

**Figure 9:**
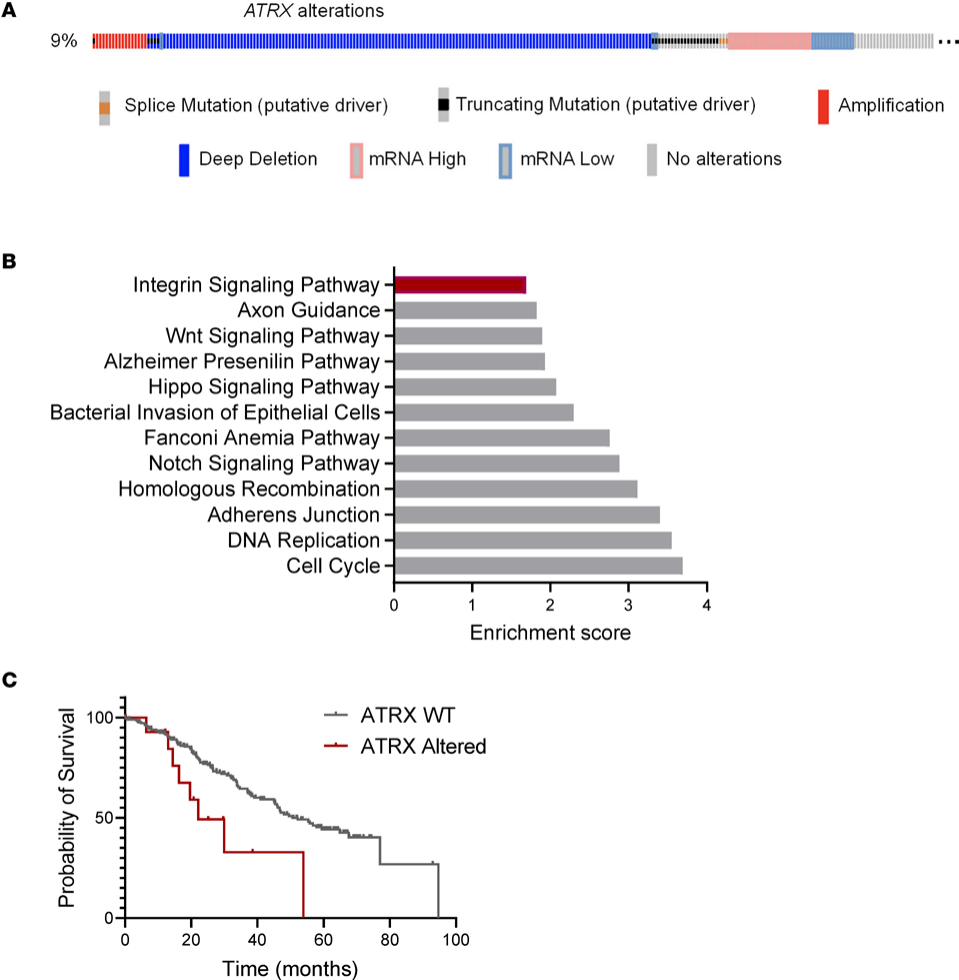
Integrin signaling is enriched in *ATRX*-altered tumors in the ICGC/TCGA Pan-Cancer Analysis of Whole Genomes dataset. (**A**) Oncoprint of *ATRX* status. *ATRX* was found to be altered in 9% of 2583 samples after “hiding mutations and copy number alterations of unknown significance” in the ICGC/TCGA Pan-Cancer Analysis of Whole Genomes dataset on cBioPortal (18, 19, 44). (**B**) Integrin signaling was enriched in the *ATRX*-altered tumors. Overrepresentation Analysis (ORA) was performed on expression data between *ATRX*-altered and unaltered groups (92). Genes submitted to ORA had higher expression in *ATRX*-altered groups than unaltered groups, and all identified genes had *q* < 0.05. The query was submitted to the Panther, KEGG, and Wikipathway cancer databases. Enrichment ratios for identified pathways with FDR < 0.05 are displayed. (**C**) Kaplan-Meier survival curve based on *ATRX* status. Hazard ratio = 3.917; 95% CI, 1.335–11.5; *P* < 0.036. Note, not all *ATRX*-altered tumors have deletions; aside from homdel, there are 2 missense mutations and 1 amplification represented in the altered group.

In order to explore whether similar cellular signaling alterations are found in human cancers with altered *ATRX* expression, we used cBioportal to examine the gene expression data of the ICGC/TCGA Pan-Cancer Analysis of Whole Genomes data set (18, 19, 44). Importantly, in line with our own experimental findings, we did find integrin signaling to be enriched in the *ATRX*-altered subset of tumors (Figure 9, A and B). Additionally, survival in the subset of patients with *ATRX*-altered tumors was significantly decreased compared with those with *ATRX* WT expression (Figure 9C). These results further support a close correlation between *ATRX* deficiency, survival, and integrin signaling.

## Discussion

Our experimental results support the hypothesis that *ATRX* loss, common in human OS, plays a key role in enhancing the aggressiveness of OS. Loss of expression of this gene increases multiple oncogenic phenotypes, including increased tumor initiation, growth, migration, invasion, and metastasis. Our investigations into the underlying cellular mechanisms driving these aggressive OS phenotypes with *ATRX* deficiency point to changes in the NF-κB pathway, ECM protein expression, ETS transcription factor binding, and integrin expression — specifically, integrins αvβ3 and αvβ5. *ATRX*-deficient cells display substantially increased sensitivity to integrin signaling inhibition. Additionally, examination of pancancer sequencing data supports these correlations between *ATRX* deficiency, survival, and integrin signaling across a range of cancer types.

NF-κB activity is known to increase tumor cell proliferation, suppress apoptosis, and promote angiogenesis (45, 46). NF-κB signaling also enhances tumor invasiveness by inducing and maintaining epithelial-mesenchymal transitions required for tumor metastasis (47). Specifically in OS, Felx et al. (48) found that NF-κB pathway activation played a central role in proliferation in the MG-63 human OS cell line, and Zhao et al. (49) reported that the NF-κB pathway was a key regulator of OS tumor growth, metastasis, and resistance to chemotherapeutics. We demonstrated increased expression of integrin β3 with *ATRX* KO in our cells. Scatena et al. (50) linked NF-κB activation with integrin αvβ3 binding to its ECM ligands. ECM components include various proteins and growth factors, such as osteopontin, fibronectin, collagens, proteoglycans, and laminins. OS cells adhere to the matrix via cell-surface receptors, primarily integrins, which bind to these ECM proteins (51). The ECM plays a critical role in tumor migration and metastasis, as tumor cells use integrin binding as well as various ECM-degrading proteases — including MMPs — to invade and metastasize (52). Several studies have examined the general interplay between these cellular pathways and OS biology. Li et al. (53) were able to inhibit OS metastasis with the combined blockade of both NF-κB signaling and integrin β1 expression in MG-63 cells. Very recently, Shi et al. (54) showed that expression of avβ3 integrins and fibronectin were both correlated with poor clinical prognosis and decreased survival in OS patients. These findings point to a precise mechanism through which OS with *ATRX* loss behave more aggressively in the clinical setting.

Our successful attenuation of xenograft tumor growth, as well as tumor cell migratory and invasive capabilities with the integrin inhibitor SB273005, supports the importance of integrin binding for increased OS aggression correlated with *ATRX* loss. In ecological contexts, it is often the case that an advantage in one environment leads to a collateral sensitivity to another environment. Given the host of adaptations and survival benefits conferred on cells with *ATRX* loss, we hypothesized that these cells would harbor some collateral sensitivity to loss of a secondary pathway. SB273005 is a potent, orally active nonpeptide integrin inhibitor with a high affinity for integrin αvβ3 (binding affinity constant, K_i_ = 1.2 nmol/L) and a somewhat lower affinity for integrin αvβ5 (K_i_ = 0.3 nmol/L) (43). Research on this inhibitor is somewhat limited to date but has included studies of its effect on bone resorption and osteoporosis, arthritis, and the production of Th2 cells and cytokine IL-10 in pregnant mice (42, 43, 55). Gomes et al. (56) studied breast adenocarcinoma cells in whole blood under flow conditions and found that a combination of SB273005 and lamifiban (a nonpeptide antagonist specific for platelet αIIbβ3) successfully inhibited adhesion to the vascular ECM. Several other αvβ3-targeting drugs have advanced to clinical trials for treatment of various solid tumors, including cilengitide, etaracizumab, and the small molecule GLPG0187 (57–62). Despite success in early clinical trials, many of these therapies did not produce clinical outcomes that were significantly improved compared with standard treatment regimens. Based on our own findings, one might ask whether a more defined target population, based on specific cellular signaling pathway alterations and gene mutations, such as *ATRX* loss, would improve these outcomes. Indeed, such a similar dependence on specific mutations for drug efficacy has been demonstrated with the use of PARP inhibitors most successfully in breast cancer tumors harboring mutations in the *BRCA1* and *BRCA2* genes (63).

In addition to altered integrin expression, we discovered increased mRNA expression of osteopontin following *ATRX* loss. Osteopontin (*SPP1*) is an important component of the ECM in bone, and high expression and secretion of osteopontin in numerous cancer types — including breast, prostate, lung, gastrointestinal, hepatocellular, cervical, and bladder cancers — have been clinically correlated with poor prognosis and shortened survival times (64–73). Gaumann et al. (74) also showed that strong expression of osteopontin correlated with progression of malignancy and metastasis in poorly differentiated sarcomas. Song et al. (75) showed that targeting osteopontin expression with miR-4262 could reduce cell invasion and migration in OS cells. In line with our study findings, binding of osteopontin to integrin αvβ3 directly activates the NF-κB pathway (50). Future assays will examine changes in the secreted proteins, including osteopontin, to further characterize how *ATRX* expression levels affect these ECM proteins.

The examination of *ATRX* binding motifs from our sequencing data shows a close similarity to ETS family transcription factor binding motifs. ETS family proteins are also involved in osteogenic differentiation and have been reported to play an important role in osteoblast development and bone formation (34, 35). Studies show that the ETS family of transcription factors plays a crucial role in driving malignancy of tumor cells by prevention of apoptosis, support of angiogenesis, and promotion of invasion and metastasis (34, 35, 76–82). ETS factors are well-known critical mediators of ECM remodeling and invasive properties in cancers, regulating a wide spectrum of ECM-related target genes (34, 83). Also fitting with our own experimental findings, ETS transcription factors are known to share crosstalk with NF-κB signaling, and ETS1 and ETS2 have been shown to bind directly to the promoter regions of integrins αv and β3, as well as their ligand, osteopontin (77, 81, 84–88).

Our research has not completely elucidated how ATRX protein expression alters ETS transcription factor activity, but the enrichment of the ETS binding motifs with changes in chromatin accessibility suggest that functional ATRX may repress these transcription factors by maintaining closed chromatin, effectively preventing the tumor-promoting sequelae that would otherwise occur with active ETS protein binding. Intriguingly, Li et al. (89) demonstrated that ETS1 and DAXX protein colocalize to the promyelocytic leukemia (PML) nuclear bodies. DAXX/Ets1-associated protein 1 (DAXX/EAP1) is able to bind to the N-terminal part of ETS1 and cause repression of transcriptional activation of ETS1 target genes, including *MMP1* and *BCL2* (89). In more recent studies, researchers have found that ATRX also localizes to the PML nuclear bodies and forms a binding complex with DAXX at this location (90, 91). Future studies should explore whether ATRX also binds with ETS1 in PML bodies, similar to DAXX/EAP1, and in this way, directly represses transcriptional activation of ETS1 target genes.

**Figure 10:**
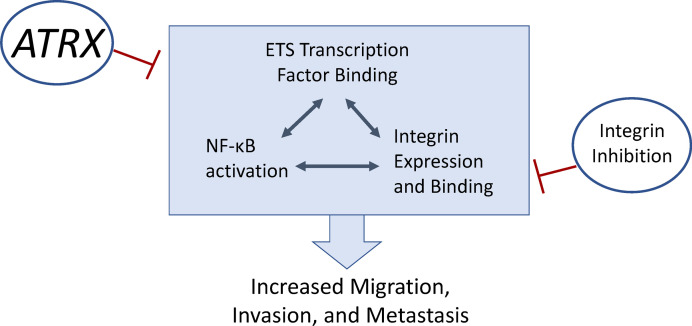
Graphical working model. Numerous studies have shown a close interaction between ETS family transcription factor binding, NF-κB pathway activation, and integrin expression and binding — all of which collectively promote increased cellular motility and metastasis in cancers. Our study has revealed that *ATRX* expression suppresses these pathways to prevent the oncogenic phenotypes of migration, invasion, and metastasis and that integrin inhibition may be a potential targeted therapy for *ATRX*-deficient OS.

As we have described, the interactions between ETS binding, NF-κB activation, integrin αvβ3 expression, and osteopontin expression have been closely linked with more aggressive tumor biology in prior studies across cancer types, but the association with *ATRX* expression has not been noted previously. This relationship may pertain to the underlying cellular signaling pathways and pathogenesis of other *ATRX*-deficient diseases, including other cancers and ATRX syndrome. In a large pancancer sequencing data set, integrin pathway signaling was enriched in this *ATRX*-mutated population, supporting the hypothesis that our findings will apply to a wide range of *ATRX*-deficient cancers. Our graphical model of these signaling pathways based upon our experimental findings is shown (Figure 10). Understanding how *ATRX* alters these cellular pathways in OS may aid in identifying new targeted therapeutics for *ATRX*-deficient OS tumors, including the potential use of current and novel integrin inhibitors. Future research should investigate further the role of ECM alterations in OS metastasis and the efficacy of integrin inhibitors as a targeted therapy for specific subsets of OS. How *ATRX* loss alters these pathways in other cancers should also be explored.

## Methods

Supplemental Methods are available online with this article.

### Data availability.

The high-throughput sequencing data from this study have been submitted to the NCBI GEO repository under accession no. GSE167546.

### Statistics.

For bar graphs, all data are presented as means ± SEM. All data were analyzed for statistically significant differences using the 1- or 2-tailed Student’s *t* test (for 2 comparisons) or ANOVA with Tukey’s multiple-comparison test. Effects over time were analyzed with repeated-measures ANOVA. Tumor-free survival curves were estimated using the Kaplan-Meier method and compared statistically using the log-rank test. JMP Pro 15.0 and/or Prism 9.0 (GraphPad Software, Inc.) were used for the statistical analyses. Any *P* value less than 0.05 was considered statistically significant.

### Study approval.

All animal studies were approved by the IACUC at Duke University.

## Author contributions

SBD, WCE, BA, and JAS conceived the project, designed experiments, and wrote the manuscript. SBD analyzed data. SBD and SHP bred, managed, and genotyped mice. Immunofluorescence was performed by HEB. SBD and DS performed experiments with the integrin inhibitor SB273005 both in vitro and in vivo. SBD, EJM, and WF performed genotyping of s.c. tumors to validate *ATRX* recombination. EJM also assisted with cross-referencing gene expression data and chromatin accessibility changes in the RNA-Seq and ATAC-Seq data. MZ and JR assisted with the common binding motif analysis. VK and SV studied growth of cells on Matrigel beds. JVS and NH analyzed sequencing data from cBioPortal. WF and DGK provided consultation on experimental design. MUS provided the graphic of tumor phenotypes of aggression.

## Supplementary Material

Supplemental data

## Version 1 09/08/2022

Electronic publication
