# Supplementary material for: Loss of ATRX promotes aggressive features of osteosarcoma with increased NF-κB signaling and integrin binding
Source: JCI Insight. 2022 Sep 8;7(17):e151583. doi: 10.1172/jci.insight.151583 (PMC9536280; doi:10.1172/jci.insight.151583)

Supplement 1

Materials and Methods

Cell Culture

143B and MG-63 human osteosarcoma cell lines as well has HEK293T cells were cultured in Dulbecco's modified Eagle's medium (Gibco; catalog #11995065) supplemented with 10% fetal bovine serum (HyClone; catalog #SH30071.03HI) and 1% penicillin-streptomycin (Gibco; catalog #15140122). The U-2 OS human osteosarcoma cell line was cultured in McCoy's 5A modified medium (Gibco; catalog #166000082) supplemented with 10% fetal bovine serum (HyClone; catalog #SH30071.03HI) and 1% penicillin-streptomycin (Gibco; catalog #15140122). All cell lines were incubated at 37°C and 5% CO2 in a humidified incubator. All cell lines were obtained from the Duke University Cell Culture Facility, which performs routine *Mycoplasma* testing and verifies cell identity by analysis of short tandem repeats. Cell line authentication was verified again after *ATRX* knockouts were created, and chromatographs are provided (Supplemental Figure 6). 143B and U-2 OS cells are from a female patient, and MG-63 cells are from a male patient.

Western blotting

Total protein was extracted from cultured cells by washing cells twice in chilled 1x Dulbecco's phosphate-buffered saline (DPBS) (Gibco; catalog #14190144) and lysing them in 1x radioimmunoprecipitation assay (RIPA) buffer (Thermo Fisher; catalog #89900) supplemented with 2x Halt protease inhibitor cocktail (Thermo Fisher; catalog #78440). Cell lysates were incubated at 4°C for 15 minutes with rocking and clarified by centrifugation at high speed in a benchtop centrifuge for 10 minutes, after which clarified lysates were incubated for 5 minutes at 95°C in 1x Laemmli gel loading buffer (Thermo Fisher; catalog #AM8546G). Lysates were

separated in 4 to 12% NuPAGE Novex Bis-Tris gels (Thermo Fisher; catalog#NP0321 or #NP0323) while in 1x NuPAGE MOPS SDS running buffer (Thermo Fisher; catalog #NP0001). Proteins were transferred onto nitrocellulose membranes (GE Healthcare; catalog #10600012) in 1x NuPAGE transfer buffer (Thermo Fisher; catalog #NP0006) at 50 V for 2 hours at 4°C. Membranes were blocked for 1 hour at room temperature or overnight at 4°C in blocking buffer (2.5 g of dried milk powder in 50 mL of TBST; TBST was made with 100 mL of 10x TBS, 10 mL of 10% Tween, with deionized water added to bring to 1L volume). Membranes were then washed three times for 5 minutes with 1x DPBS (Gibco; catalog #14190144) with 0.05% Tween 20 (PBS-T). Primary antibodies (Anti-ATRX: Cell Signaling Technology #14820; Anti-α-tubulin: Abcam #ab7291) were diluted in blocking buffer (1:1000 for ATRX, and 1:5000 for α-Tubulin). These antibodies were added to the membranes, incubated for 1 hour at room temperature or overnight at 4°C, washed three times for 5 minutes in 1x DPBS (Gibco; catalog #14190144) with 0.05% Tween 20 (PBS-T), and the incubated for 1 hour at room temperature with IRDye-coupled goat anti-rabbit 800CW (LI-COR; catalog #925-32211) or goat anti-mouse 680RD (LI-COR; catalog #925-68070) secondary antibodies diluted 1:20,000 in blocking buffer. Images were captured on an Odyssey Fc imager (LI-COR).

RT-qPCR

RNA was extracted from cultured cells following the protocol of the ReliaPrep RNA Cell Miniprep System (Promega; catalog #Z6012). Concentrations of the RNA extractions were measured with a Thermo Fisher NanoDrop One spectrophotometer, and all RNA samples were diluted to equal the sample with the lowest concentration. The Applied Biosystems High-Capacity cDNA Reverse Transcription kit (Applied Biosystems; catalog #4374966) was used for reverse transcription. Samples were incubated in a thermocyler at 25°C for 10 minutes, 37°C for

2 hours, 85°C for 5 minutes, and then brought to 4°C. Final cDNA samples were diluted 1:5 with molecular grade water (Invitrogen; catalog #10977015). qPCR was performed in Applied Biosystems MicroAmp Fast Optical 96-well barcoded reaction plates (Thermo Fisher; catalog #4346907). Samples were added to 10 uM forward and reverse primers and 2x KAPA SYBR Fast Universal qPCR Master Mix (Kapa Biosystems; catalog #KK4602). Primers for the housekeeping gene, *HPRT1*, were as follows: forward 5'-GAA AAG GAC CCC ACG AAG TGT-3' and reverse 5'-AGT CAA GGG CAT ATC CTA CAA-3'. Primers for *ATRX* were as follows: forward 5'- TCC TTG CAC ACT CAT CAG AAG AAT C-3' and reverse 5'- CGT GAC GAT CCT GAA GAC TTG G-3'.

shRNA knockdown

The *ATRX* shRNA clones TRCN0000013588, TRCN0000013589, TRCN0000013590, TRCN0000013591, and TRCN0000013592 were obtained from Sigma Mission shRNA collection (https://www.sigmaaldrich.com/life-science/functional-genomics-and-rnai/shrna/individual-genes.html). A non-silencing PLKO-GFP plasmid also was obtained from the same Sigma Mission shRNA collection (catalog #SHC005). HEK293T cells were plated at 500,000 cells/well of a 6-well culture plate. Twenty-four hours later, for each well of cells, lentiviral packaging plasmids (1.8 µg pΔ8.9 and 0.2 µg pCMV-VSV-G (Addgene; catalog#8454)) were combined with 2 µg shRNA plasmid in 200 µL Opti-Mem (Thermo Fisher; catalog #11058021) in a centrifuge tube. In a corresponding centrifuge tube, 4 µL Lipofectamine 2000 (Invitrogen; catalog #11668019) was added to 200 µL Opti-Mem. The plasmid mixture was added to the Lipofectamine mixture and incubated for 20 minutes at room temperature. The wells of HEK293T cells were washed with 1x DPBS (Gibco; catalog #14190144) and then 800 µL Opti-Mem was added to each well. The 400 µL mixture of

plasmids and Lipofectamine were then added to each well. Plates were incubated at 37°C and 5% CO2 in a humidified incubator for 3 hours. Then, the wells were aspirated and washed with 1 mL DPBS before adding 2 mls of Dulbecco's modified Eagle's medium (Gibco; catalog #11995065) supplemented with 10% fetal bovine serum (HyClone; catalog #SH30071.03HI) and 1% penicillin-streptomycin (Gibco; catalog #15140122), and plates were returned to the incubator. Target 143B cells were plated 24 hours later at 500,000 cells/well of a 6-well culture plate, and after another 24 hours, viral media was removed from the HEK293T cells with a 10 mL syringe, filtered through a 0.45 μm Polyethersulfone Membrane (VWR; catalog #28145-505) into a 15 mL conical tube containing 4 μL Polybrene. The HEK293T cells received fresh complete media, and each target 143B well was aspirated before adding the viral media and continuing the incubation. This viral media transfer was repeated 24 hours later, and another 24 hours later, cells were passaged and moved into 10 cm round tissue culture dishes. 3 μL of puromycin at 2 mg/mL was added to each culture dish to select for cells with successful shRNA plasmid uptake. Each plasmid was allowed to multiply to confluence in a T75 plate, and then clones were tested for *ATRX* expression by both qPCR and Western blotting to confirm *ATRX* knockdown. The two strongest knockdowns were used with the non-silenced control for subsequent experiments.

***CRISPR-Cas9 knockout of* ATRX**

sgRNA sequences targeting exon 4 of *ATRX* were designed using ChopChop (1) and CasOffinder (2) and cloned into the px459 vector from Feng Zhang, for coexpression of a sgRNA with S. pyogenes Cas9 and a puromycin resistance marker (Addgene plasmid #62988). The sgRNA sequences used for generating the knockout cell lines are CAGGATCGTCACGATCAAAG (ATRX-3) and TCGTGACGATCCTGAAGACT (ATRX-4)

and are designed to generate a 20 bp deletion when used together. MG-63 or 143B knockout cells were created by cotransfection of ATRX-3 and ATRX-4 sgRNA vectors using TransIT-LT1 (Mirus) according to manufacturer's suggestions, followed by transient selection with puromycin. Surviving cells were expanded and re-plated into 96 well plates at limiting dilution to isolate clonal lines. Clones were screened for CRISPR-mediated deletion by loss of a Tsp45I restriction site and positive clones were Sanger sequenced to confirm and characterize the genomic deletion event. Successful knockout of *ATRX* was also verified by Western blot and RT-qPCR.

Luciferase labelling of cells

143B WT and *ATRX* CRISPR-Cas9 knockout cells were transfected with the pLenti CMV Puro LUC plasmid (Addgene #17477) following the same transfection protocol as for the shRNA plasmids described above. Following puromycin selection of all transfected cells, each LUC-labelled cell line was passaged and plated at 500 cells/plate in 10-cm tissue culture plates. After about one week, single colonies of cells were harvested by gently scraping the individual clone's colony in the plate with a 200 uL pipet tip and then transferring to a single well of a 24-well cell culture plate. Multiple colonies were collected in this way and then each isolated clone was allowed to expand to confluence. The Dual-Glo Luciferase Assay (Promega; catalog #E2920) was then performed and luciferase fluorescence levels were read on the SpectraMax plate reader to identify individual WT and KO clones that expressed equal levels of luciferase (Supplemental Figure 2D). The two most similar clones were then expanded for use in the orthotopic injections described below.

Growth of cells on Matrigel bed

100 μl of Matrigel (Corning, 354230) was evenly spread in each well of a pre-chilled 24-well plate. The plate was placed in an incubator for 15 minutes to allow the Matrigel to set. Then, 5 x 10^3 cells per well were seeded onto the Matrigel bed. Cells were imaged every 24 hours using the ZEISS AxioVert microscope.

In vitro immunofluorescent staining of cells

Coverslips (Electron Microscopy Cat. #72230-01) stored in 90% EtOH were placed on parafilm in a petri dish and washed 3x with sterile 1X PBS. Coverslips were coated with 10ug/mL human fibronectin for 1hr at 37°C then gently washed 3x with 1XPBS. Coverslips were then carefully transferred to 24-well plate (with #5 watchmaker's tweezers), and cells were plated at ≤50% cell density and allowed to spread for 12 to 24 hours. Media was removed and coverslips were gently washed once with 1XPBS at room temperature. Cells were fixed with cold 4% PFA for 10 minutes then washed 2-3x with 1XPBS. Coverslips were moved to staining chamber (p1000 tip box with parafilm and lid) and 1XPBS was quickly added. To permeabilize, PBS was aspirated off and 0.1% Triton-X-100 in PBS was added for 5 minutes at room temperature. Coverslips were then washed 3x 5 minutes with 200ul 1XPBS. Coverslips were blocked with 5% BSA for 30minutes. Primary antibodies (Alexa Fluor 488 Phalloidin, Cell Signaling, cat#8788; Integrin β3, Thermo Fisher, cat#13166) were diluted 1:200 in 1% BSA in PBS, and cells were stained either 1 hour at room temperature or overnight at 4°C in humidified chamber. Antibodies were aspirated and coverslips were washed 3x 5minutes with 1XPBS. Secondary antibodies (Thermo Fisher, cat#A10523) were added at 1:1000 in 1% BSA for 1 hour at room temperature. Secondary antibodies were aspirated and coverslips were washed 3x 5minutes with 1XPBS. Coverslips were gently inverted, with excess PBS removed, mounted onto slides with mounting media containing DAPI (Thermo Fisher, cat#P36931) and sealed with nail polish.

Imaging was performed on a Leica SP5 Confocal Laser Scanning Microscope. Images were captured with a 40X oil objective. Images were analyzed using ImageJ (3).

Scratch wound assays

143B NS and shATRX cells were plated in separate wells of a 96-well culture plates at 2 x 10^4 cells/well in 200 uL of culture media. After 24 hours, cells had reached confluence. A sterile 20 uL pipet tip was manually scratched across each well to create the scratch wound. Wells were aspirated to remove floating cells and replaced with 200 uL of culture media. The plate was placed in the Incucyte Live-Cell Imaging System (Essen BioScience), and images were collected hourly for 48 hours. Five wells of NS and 6 wells of each shATRX with clean scratch wounds were analyzed. Images were downloaded and analyzed by ImageJ (3). The MG-63 WT and KO cells were plated in the same way, however for these cells, we used the Woundmaker™ (Essen BioScience) to create the scratch wounds in each well. Thirteen scratch-wound wells of WT and 10 wells of KO were analyzed in the same way as with the 143B cell line.

Transwell migration and Boyden invasion assays

The Matrigel-coated invasion assay plate (Corning; catalog #354480) was removed from the foil bag and allowed to come to room temperature. Culture media consisting of DMEM and 1% Pen Strep, but no FBS, was also brought to room temperature, and then 500 µL of this media was added to each Matrigel-coated well to hydrate the inserts while incubating at 37°C for 4 hours. At this time, 1% BSA was added to 50 mLs of the DMEM culture media. The uncoated plate (Corning; catalog #354578) for the migration assay was removed from its foil bag. In each plate's upper inserts, the 143B WT and KO cell lines were plated at 10,000 cells per well and the MG-63 WT and KO cell lines were plated at 20,000 cells per well in 200 µL

of the DMEM with 1% BSA. Then 750 µL of DMEM with 1% Pen Strep and 10% FBS was placed in the bottom well below each insert. All plates were incubated at 37°C. The migration assay plates were removed after 18 hours and the Matrigel-coated plates were removed after 41 hours. Media was aspirated from the upper and lower chambers of each well and washed twice with 1x PBS. Cells were fixed with 4% paraformaldehyde for 5 minutes and then washed again with 1x PBS. Cells were then permeabilized with 750 µL Perm buffer (1x PBS with 0.2% Triton-X-100) and incubated on bench top for 20 minutes. Then the cells were washed again with 1X PBS and stained with 0.1% Crystal Violet for 20 minutes on bench top. Once again, cells were washed with 1x PBS and then non-migrated cells were scraped off the inside of each insert with a sterile cotton swab (VWR; catalog #149-0332). Inserts were allowed to dry for at least one hour. A scalpel was used to cut out each insert and mount to a microscope slide with Cytoseal (Thermo Scientific; catalog #83104) and a coverslip. Slides were dried for 3 hours and then imaged on a light microscope, recording five high powered fields per membrane. For the 143B cell line, 8 wells of WT and 8 wells of KO were analyzed for both the uncoated and Matrigel-coated assays. For the MG-63 cell line, 12 wells of WT and 12 wells of KO were analyzed for both uncoated and Matrigel-coated assays. Images were analyzed using ImageJ (3) to get total cell counts per slide.

Osterix-Cre *mouse model experiment*

The conditional osteoblast-specific mouse model builds from a previously-established (4, 5) transgeneic *Osterix-Cre* (*Osx-Cre*) mouse model with conditional (floxed) alleles of both *p53* ($p53^{fl/fl}$) and *Rb* ($Rb^{fl/fl}$) and with a Tet-off cassette providing an additional level of temporal control. Female *Osx1-GFP::Cre* mice (mixed background) were kindly donated by the Courtney Karner lab (UT Southwestern, Dallas, TX), and male $Rb^{fl/fl}p53^{fl/fl}$ mice (mixed background) were

donated by the Anton Berns lab (The Netherlands Cancer Institute, Amsterdam, The Netherlands). Male *Atrx*$^{fl/y}$ mice (mixed background) were kindly donated by the Richard Gibbons lab (University of Oxford, Oxford, England). These mice were crossed to generate experimental cohorts of males and females that were either *Osx-Cre+p53*$^{fl/fl}$*Rb*$^{fl/fl}$ (n=26 males and 25 females) or *Osx-Cre+p53*$^{fl/fl}$*Rb*$^{fl/fl}$*Atrx*$^{fl/fl/y}$ (n=22 males and 25 females). Mice were fed a rodent diet supplemented with doxycycline until weaning (Envigo; catalog #TD.120769), upon which we removed the doxycycline diet and monitored mice of each genotype for tumor development. To more comprehensively identify tumors that developed in any bone and to further increase our sensitivity in tumor detection, we performed monthly fluoroscopy on a subcohort of 10 females and 10 males of each genotype (Supplemental Figure 1A). Tumors were collected, locations were noted, and gene recombination of tumors was confirmed by PCR to distinguish the knockout allele from the floxed (unrecombined) and wildtype allele (Supplemental Figures 1B).

Subcutaneous xenografts for tumor growth comparison

For xenograft studies, SCID-beige mice (strain code 250) were obtained from Charles River Laboratories. For each cell line, we injected 1.0 x 10^6 cells suspended in 200 µL saline subcutaneously into the flank of mice when they were 8 to 10 weeks old. For the shRNA/NS cells, there were three males and two females per treatment group, with three same-sex mice per cage and one mouse per treatment group (randomly chosen) in each cage. For the WT/KO cells, there were five males and five females per treatment group, with five same-sex mice per cage and two mice receiving one cell type and three mice receiving the other cell type (randomly chosen) per cage. Resultant tumors were measured every two to three days with calipers to compare growth rates. When the tumors grew to the point of humane endpoints (defined as

respiratory changes, greater than fifteen percent body weight loss, dehydration, decreased physical activity, or large tumor burden), mice were sacrificed. Final tumor volume and mass were measured.

Xenograft tumor immunohistochemistry

Xenograft tumors were harvested, formalin-fixed, and paraffin-embedded. The Duke Histopathology Core Research Group prepared slides and performed H&E and immunohistochemistry (IHC) staining for Ki-67 (Thermo Fisher, catalog#RM-9106-S), ATRX (Abcam, catalog#ab270272), and cleaved caspase 3 (Cell Signaling, catalog#9661S). IHC staining was interpreted and quantified with the help of a board-certified veterinary pathologist.

Orthotopic mouse model of metastasis-injections, amputations, IVIS imaging

The LUC-labelled 143B WT and CRISPR KO cell lines described below were prepared at a concentration of 6.0 x 10^5 cells suspended in 30 μL 50:50 saline:Matrigel solution (Corning; catalog#354234). Cells were injected into the subperiosteal space of the tibia in 8- to 10-week-old SCID-beige mice (obtained from Duke University's Division of Laboratory Animal Resources (DLAR) breeding core facility) (WT: n=5 males and 10 females, KO: n=6 males and 11 females). When tumors were 1 cm^3 in size, the mice underwent amputation of the affected limb. Luciferase luminescence was measured with the IVIS imaging device immediately after injection and then every 2 to 3 days to compare metastasis burden and disease progression in our mice. For each imaging session, mice were anesthetized and then injected intraperitoneally with 100 μL of D-luciferin at 28.6 mg/mL (Gold Biotechnology; catalog #LUCNA-1G). IVIS images were collected sequentially for 30 minutes to capture peak luciferin tissue distribution. Luminescence readings were collected for the lung field

region of interest for each mouse and compared to analyze metastatic burden in the lungs. The data shown in Figure 3K were gathered at one-week post-amputation.

RNA Sequencing

RNA-Seq was performed on the *ATRX* knockdown or non-silenced 143B human OS cell lines in collaboration with the Duke Center for Genomic and Computational Biology. RNA-Seq data was processed using the TrimGalore toolkit which employs Cutadapt (6) to trim low quality bases and Illumina sequencing adapters from the 3' end of the reads. Only reads that were 20nt or longer after trimming were kept for further analysis. Reads were mapped to the GRCh37v75 version of the human genome and transcriptome (7) using the STAR RNA-Seq alignment tool (8). Reads were kept for subsequent analysis if they mapped to a single genomic location. Gene counts were compiled using the HTSeq tool. Only genes that had at least ten reads in any given library were used in subsequent analysis. Normalization and differential expression were carried out using the DESeq2 Bioconductor package (9, 10) with the R statistical programming environment. The false discovery rate was calculated to control for multiple hypothesis testing. PCA results from sequencing samples are shown Supplemental Figure 5B. Gene set enrichment analysis (11) was performed to identify differentially regulated pathways and gene ontology terms for each of the comparisons performed.

ATAC Sequencing

ATAC-Seq data was processed using the TrimGalore toolkit (http://www.bioinformatics.babraham.ac.uk/projects/trim_galore) which employs Cutadapt (6) to trim low quality bases and Illumina sequencing adapters from the 3' end of the reads. Only reads that were 20nt or longer after trimming were kept for further analysis. Reads were mapped to the hg19 version of the human genome using the bowtie alignment tool (12). Reads were kept for

subsequent analysis if they mapped to a single genomic location. Amplification artifacts were removed using the Picard toolkit (http://broadinstitute.github.io/picard). Regions of open chromatin regions were called using the MACS2 peak calling algorithm (13). The peaks were set to a 300nt window surrounding the mode location. Peaks that overlapped across the two conditions being compared were merged into a single peak. The number of reads from each individual sample that overlapped the peaks were quantified. Normalization and differential openness of chromatin regions were calculated using the DESeq2 Bioconductor package (9, 10) with the R statistical programming environment. Peaks were then annotated with their nearest gene using the GRCh37v758 of the human transcriptome. Quality control statistics are shown in Supplemental Figure 5C.

Chromatin binding motif analysis

To understand ATRX chromatin binding, chromVAR was applied to analyze the ATAC-Seq data for variability of DNA motif enrichment in the accessible regions (14). In brief, chromVAR aggregates accessible regions sharing the same DNA motif, then compares the observed accessibility of all peaks containing that motif with a background set of peaks normalized for known technical confounders. The aligned fragments and peaks were used as inputs. For reading in peaks in the narrowpeak format, peaks were first resized to width = 500 bp and then in case of overlapping peaks, the peak with strongest signal was retained. In addition, peaks were filtered such that each peak should have at least one fragment across all the samples. To determine background peaks the GC content was used and hg19 genome sequence was selected as input. To identify significantly variable motifs among cells chromVAR scans the peaks for binding motif occurrences, using a curated collection of motifs provided from “motifmatchr” package (15). In addition to curated binding motifs, peaks were also scanned for all 6-mers. Cell-cell

similarity was visualized in a two-dimensional t-SNE plot using the bias-corrected deviations in accessibility for both curated motifs and 6-mers.

Nuclear extractions and NF-κB ELISA

143B and MG-63 wildtype and *ATRX* knockout cells were plated at 1 x 10^7 cells per culture dish in two 10 cm culture dishes for each cell type. After 24 hours of incubation, cells were harvested and nuclear extractions were performed following the protocol of the Active Motif Nuclear Extract Kit (catalog #40010). Final extracted protein concentrations for each cell type were measured using a Bradford protein assay. The Active Motif TransAm NFKB Family kit (catalog #43296) was used to perform the ELISA assay with our nuclear extracts following the kit's protocol. Recombinant p50 protein (Active Motif, catalog #31101) was used for the standard curve. Absorbance measurements were read using the SpectraMax i3x spectrophotometer.

Bioactives compound screen

MG-63 wildtype and *ATRX* knockout cells were screened in triplicate with the Bioactive Compound Library (catalog #L1700) from Selleckchem, a diverse collection of 2100 compounds with demonstrated bioactivity. Library compounds were plated onto 384 well tissue culture plates using a Labcyte Echo for a final concentration of 1 uM after addition of 750 cells in 50 ul media using a Matrix WellMate. Cells were incubated with drug at 37°C for 72 hours and then assayed for viability using Cell Titer-Glo (Promega; catalog #G7571). Cell viability for each well was normalized against the DMSO wells contained in each plate. All screens were performed in the Duke Functional Genomics Shared Resource.

IC^{50} assays

We further validated the integrin inhibitor SB273005 (Selleckchem; catalog #S7540) from the drug screen using IC^{50} assays. MG-63 wildtype and knockout cells were plated at 10,000 cells

per well in 96-well plates with 100 uL DMEM. Cells were incubated overnight. Serial dilutions of drug or DMSO vehicle control were prepared to test plated concentrations of 1,000 nM, 200 nM, 40 nM, 8 nM, 1.6 nM and 0.32 nM drug. After incubation for 24 hours, 80 uL Cell Titer-Glo reagent (Promega, catalog #G7571) was added to each well using a multichannel pipette. The plate was wrapped in aluminum foil and placed on an orbital shaker for five minutes. Then, the plate was imaged on the SpectraMax i3x spectrophotometer at ten minutes, thirty minutes, and one hour.

In vivo study with integrin inhibitor SB273005

The integrin-inhibitor SB273005 (Selleckchem; catalog #S7540) was tested in vivo with xenograft mouse tumors to further validate its efficacy as a therapeutic for *ATRX*-deficient OS. We used the U-2OS human OS cell line (ATRX-null) to create subcutaneous tumors in NSG mice (obtained from Duke University's DLAR rodent breeding core facility), injecting 1.0 x 10^6 cells in 200 μL of a 50:50 saline:Matrigel solution subcutaneously in the flank. Each cohort contained five male and five female mice at 8 to 10 weeks of age. As tumors became palpable, mice were randomly assigned to treatment with a vehicle control or SB273005 at a dose of 50 mg/kg/day, a dose well-tolerated and therapeutically effective in rats in a study by Badger et al. (16). Tumor volume was monitored with caliper measurements every two to three days for a thirty-day treatment course.

In vitro assays with integrin inhibitor SB273005

Cell Titer-Glo fluorescence assays of cell viability were repeated, and a dose of 8 nM was determined to be the highest dose of SB273005 (Selleckchem; catalog #S7540) that could be administered to cells in vitro with minimal differential cell survival between wildtype and knockout MG-63 cells. Cells were plated and treated with SB273005 or DMSO (vehicle) alone

at the 8 nM dose, and in vitro scratch wound assays and migration and invasion chamber assays were repeated as previously described, but with four treatment groups: wildtype cells treated with vehicle control, wildtype cells treated with integrin inhibitor, knockout cells treated with vehicle control, and knockout cells treated with integrin inhibitor.

Analysis of the ICGC/TCGA Pan-Cancer Analysis of Whole Genomes sequencing dataset

cBioPortal was used to compare *ATRX*-altered versus *ATRX*-unaltered tumors in the ICGC/TCGA Pan-Cancer Analysis of Whole Genomes dataset (17-19). An oncoPrint analysis was perfomed on the cBioPortal query while filtering for mutations and copy number alterations of known significance. In the (n=21) altered group, two patients have missense mutations, one patient has an amplification, and the remaining patients have *ATRX* deletions. Over-representation Analysis (ORA) was then performed on expression data between *ATRX*-altered and unaltered groups (20). Genes submitted to ORA had higher expression in *ATRX*-altered groups than unaltered groups, and all identified genes had q values < 0.05. The query was submitted to the Panther, KEGG, and Wikipathway cancer databases.

Quantification and Statistical Analysis

For bar graphs, all data are presented as means +/- SEM. All data were analyzed for statistically significant differences using the Student's t-test (for two comparisons) or analysis of variance with Tukey's multiple comparisons test (for multiple comparisons). Effects over time were analyzed with repeated-measures analysis of variance. Tumor-free survival curves were estimated using the Kaplan-Meier method and compared statistically using the log-rank test. Next generation sequencing statistical analysis is described in the RNA-Seq and ATAC-Seq methods section. JMP Pro 15.0 and/or Prism 9.0 (GraphPad Software, Inc.) were used for the statistical analyses. Any p value less than 0.05 was considered statistically significant.

References:

1. Montague TG, et al. CHOPCHOP: a CRISPR/Cas9 and TALEN web tool for genome editing. *Nucleic Acids Res*. Jul 2014;42(Web Server issue):W401-7.

2. Bae S, et al. Cas-OFFinder: a fast and versatile algorithm that searches for potential off-target sites of Cas9 RNA-guided endonucleases. *Bioinformatics*. May 15 2014;30(10):1473-5.

3. *ImageJ*. U. S. National Institutes of Health, Bethesda, MD, USA; 1997-2018. https://imagej.nih.gov/ij/

4. Berman SD, et al. Metastatic osteosarcoma induced by inactivation of Rb and p53 in the osteoblast lineage. *Proc Natl Acad Sci U S A*. Aug 19 2008;105(33):11851-6.

5. Walkley CR, et al. Conditional mouse osteosarcoma, dependent on p53 loss and potentiated by loss of Rb, mimics the human disease. *Genes Dev*. Jun 15 2008;22(12):1662-76.

6. Martin M. CutAdapt removes adapter sequences from high-throughput sequencing reads. *Bioinformatics in action*. 2011;17:10-12.

7. Kersey PJ, et al. Ensembl Genomes: an integrative resource for genome-scale data from non-vertebrate species. *Nucleic Acids Res*. Jan 2012;40(Database issue):D91-7.

8. Dobin A, et al. STAR: ultrafast universal RNA-seq aligner. *Bioinformatics*. Jan 1 2013;29(1):15-21. doi:10.1093/bioinformatics/bts635

9. Love MI, et al. Moderated estimation of fold change and dispersion for RNA-seq data with DESeq2. *Genome Biol*. 2014;15(12):550. doi:10.1186/s13059-014-0550-8

10. Huber W, et al. Orchestrating high-throughput genomic analysis with Bioconductor. *Nat Methods*. Feb 2015;12(2):115-21. doi:10.1038/nmeth.3252

11. Mootha VK, et al. PGC-1alpha-responsive genes involved in oxidative phosphorylation are coordinately downregulated in human diabetes. *Nat Genet*. Jul 2003;34(3):267-73. doi:10.1038/ng1180

12. Langmead B, et al. Ultrafast and memory-efficient alignment of short DNA sequences to the human genome. *Genome Biol*. 2009;10(3):R25. doi:10.1186/gb-2009-10-3-r25

13. Zhang Y, et al. Model-based analysis of ChIP-Seq (MACS). *Genome Biol*. 2008;9(9):R137. doi:10.1186/gb-2008-9-9-r137

14. Schep AN, et al. chromVAR: inferring transcription-factor-associated accessibility from single-cell epigenomic data. *Nat Methods*. Oct 2017;14(10):975-978. doi:10.1038/nmeth.4401

15. *motifmatchr: Fast Motif Matching in R.* R package version 1.12.0; 2020.

16. Badger AM, et al. Disease-modifying activity of SB 273005, an orally active, nonpeptide alphavbeta3 (vitronectin receptor) antagonist, in rat adjuvant-induced arthritis. *Arthritis Rheum*. Jan 2001;44(1):128-37. doi:10.1002/1529-0131(200101)44:1<128::AID-ANR17>3.0.CO;2-M

17. Cerami E, et al. The cBio cancer genomics portal: an open platform for exploring multidimensional cancer genomics data. *Cancer Discov*. May 2012;2(5):401-4. doi:10.1158/2159-8290.CD-12-0095

18. Gao J, et al. Integrative analysis of complex cancer genomics and clinical profiles using the cBioPortal. *Sci Signal*. Apr 2 2013;6(269):pl1. doi:10.1126/scisignal.2004088

19. Consortium ITP-CAoWG. Pan-cancer analysis of whole genomes. *Nature*. Feb 2020;578(7793):82-93. doi:10.1038/s41586-020-1969-6

20. Liao Y, et al. WebGestalt 2019: gene set analysis toolkit with revamped UIs and APIs. *Nucleic Acids Res*. Jul 2 2019;47(W1):W199-W205. doi:10.1093/nar/gkz401

Supplemental Figure 1: Loss of *ATRX* expression increased the rate of tumor formation in an *Osterix-Cre* driven mouse model of OS.

A) Examples of fluoroscopic imaging to detect tumors in the Osx-Cre driven mouse model. Top: This male *ATRX*-floxed mouse developed an OS of the distal femur at 3 months post-weaning. This tumor was not palpable on physical examination at this stage but was detectable by fluoroscopy. One month later, this tumor had grown and was now detectable on physical examination as well. Bottom: This female *ATRX*-WT mouse developed an OS of the proximal humerus at 5 months post-weaning. Again, this tumor was detected by fluoroscopy, but was first palpable on physical examination one month later. B) Genotyping was used to validate *ATRX* recombination or wildtype status with DNA collected from a subset of mouse tumors with matched skin samples. Primers used were PPS 1.17 5'-AAC TCA TTC AAC TGC CC-3' and PPS 1.28 5'-CAT TTA ATC CCT CCT GCC-3'. WT band at 1400 bp, ATRX floxed band at 1600 bp, and ATRX-NEO recombined band at 600 bp.

Supplemental Figure 2:

A) Aggressive tumor cellular phenotypes, including tumor initiation, tumor growth, and migration, invasion and metastasis. B) Sanger sequencing results showing effective CRISPR-Cas9 knockout of *ATRX* expression in the 143B human OS cell line and the MG-63 human OS cell line. C) All 143B cell types (NS and shRNA KDs) showed high cellular proliferation with no significant difference in measure of percent confluence over time detected with the Incucyte Live Cell Imager. D) Comparison of average luminescence in individual 143B WT and KO clones transfected with the LUC plasmid. WT clone 6 and KO clone 1 were similar in luminescence levels, so these were used for the orthotopic injection experiment.

Supplemental Figure 3:

A) and B) Notable differences in morphological appearances (specifically, increased branching networks between cells) were apparent between WT and KO cells in the slower-growing MG-63 cell line after 72 hours (A) and 96 hours (B) Yellow arrows highlight the increased branching networks forming between KO cells relative to WT cells at 72 hours C) MG-63 *ATRX* KO cells show significantly increased expression of integrin β3 compared to WT cells (Student's t-test, $p<0.0001$, KO n=43, WT n=35). D) RNA-Seq gene track for osteopontin (*SPP1*) showing increased mRNA expression with ATRX KD (log fold change over NS control of 1.3 and 2.52 for shATRX-1 and shATRX-2, respectively). Osteopontin is one of the key matrix components to which integrins αvβ3 bind, and expression of osteopontin has been correlated with poor survival in patients with OS. E) Representative images of KO+Vehicle compared to KO+Drug scratch wounds at three time points in the MG-63 cell line treated with 8nM SB273005 or vehicle control (DMSO).

Supplemental Figure 4: Enrichment plots for GSEA pathways altered with *ATRX* KD.

A) Enrichment plots for several of the most upregulated pathways found by GSEA (Curated Pathways). Many of these pathways were associated with NF-κB signaling. B) Enrichment plots for several of the most downregulated pathways found by GSEA (Curated Pathways). Many of these pathways are related to ECM remodeling.

Supplemental Figure 5:

A) Manhattan plot of ATAC-Seq regions of differential chromatin openness for each shRNA compared to non-silenced control. These data support a global genomic role of *ATRX* as a chromatin remodeler. B) PCA results as quality check of samples used for RNA sequencing. C) Quality control statistics for ATAC-Seq analysis.

Supplemental Figure 6:

Chromatographs verifying cell line authenticity for all cell lines used in this manuscript

Supplemental Figure 1

A Male *Osx-Cre+ p53*$^{fl/fl}$*Rb*$^{fl/fl}$*ATRX*$^{fl/y}$

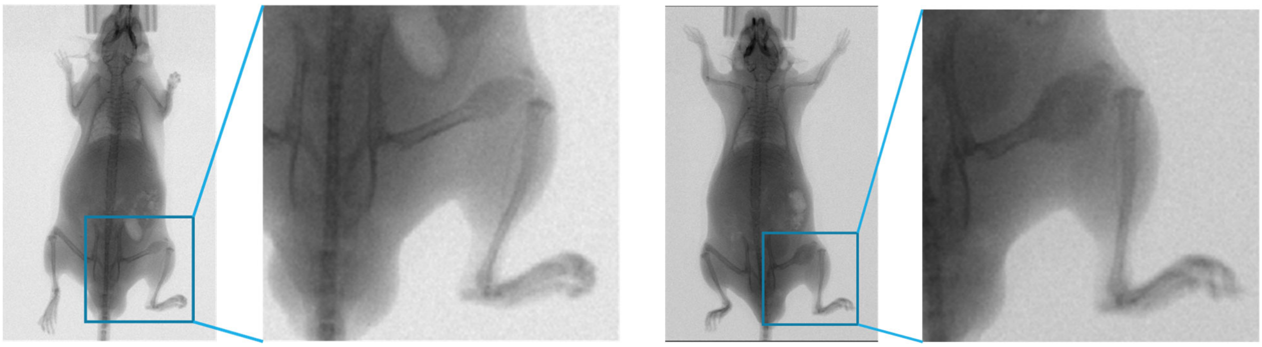

3 months post-weaning | 1 month later

Female *Osx-Cre+ p53*$^{fl/fl}$*Rb*$^{fl/fl}$*ATRX*$^{WT/WT}$

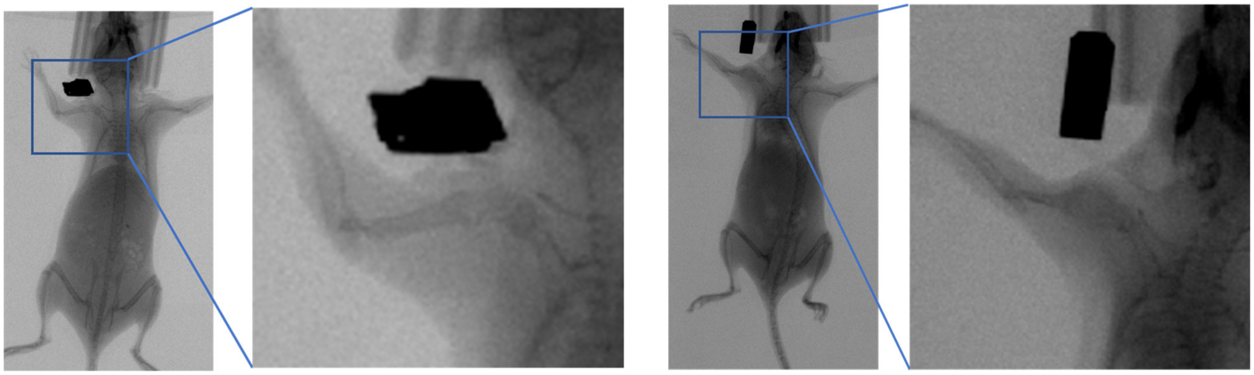

5 months post-weaning | 1 month later

B

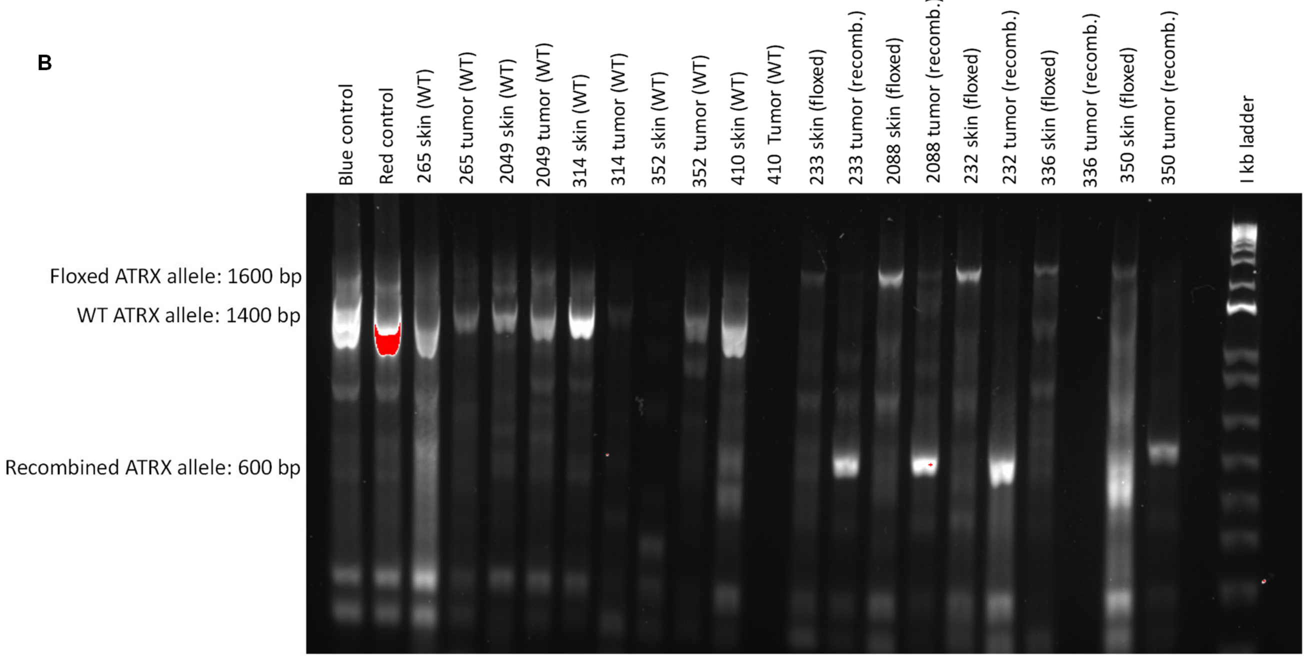

Supplemental Figure 2

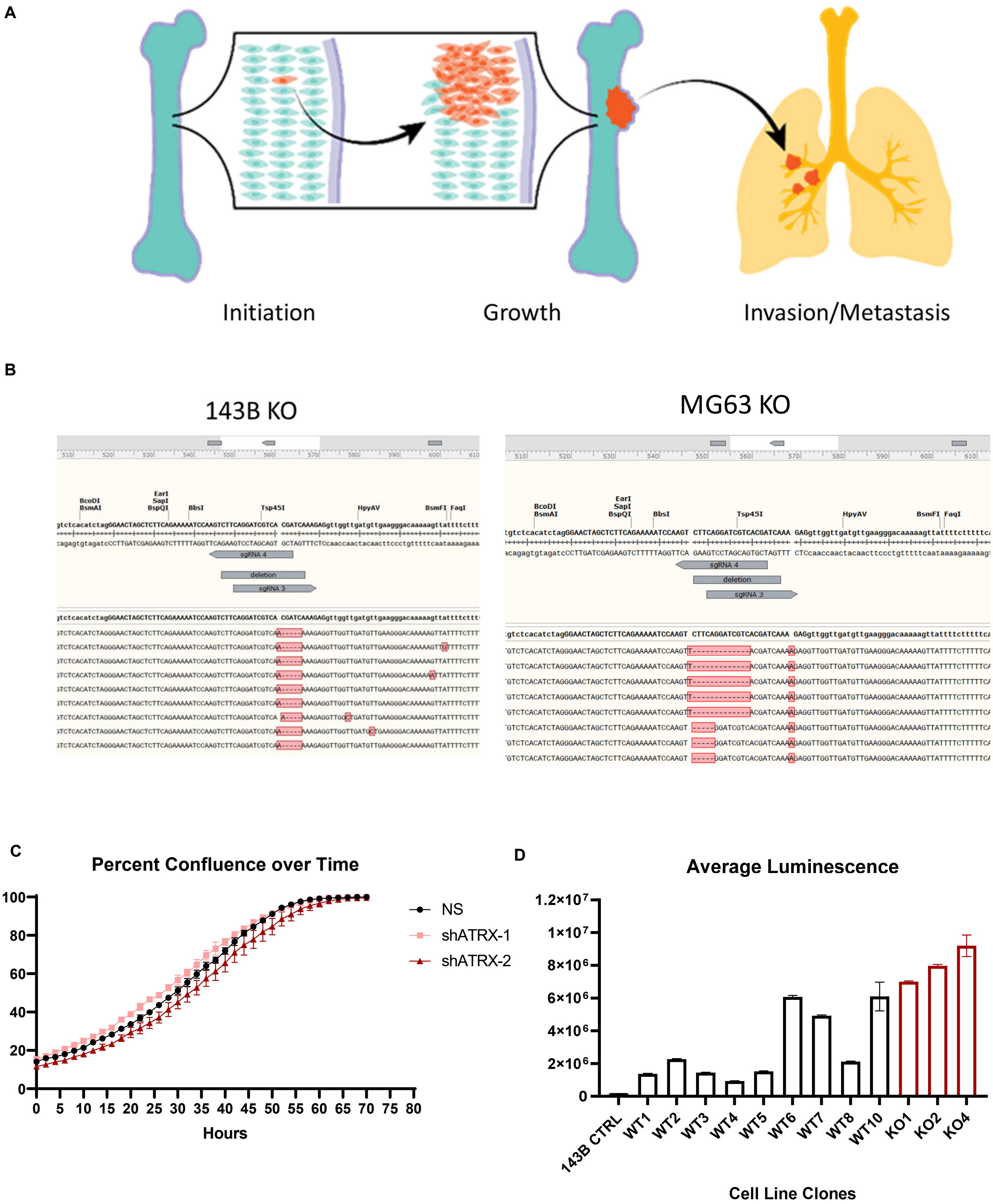

Supplemental Figure 3

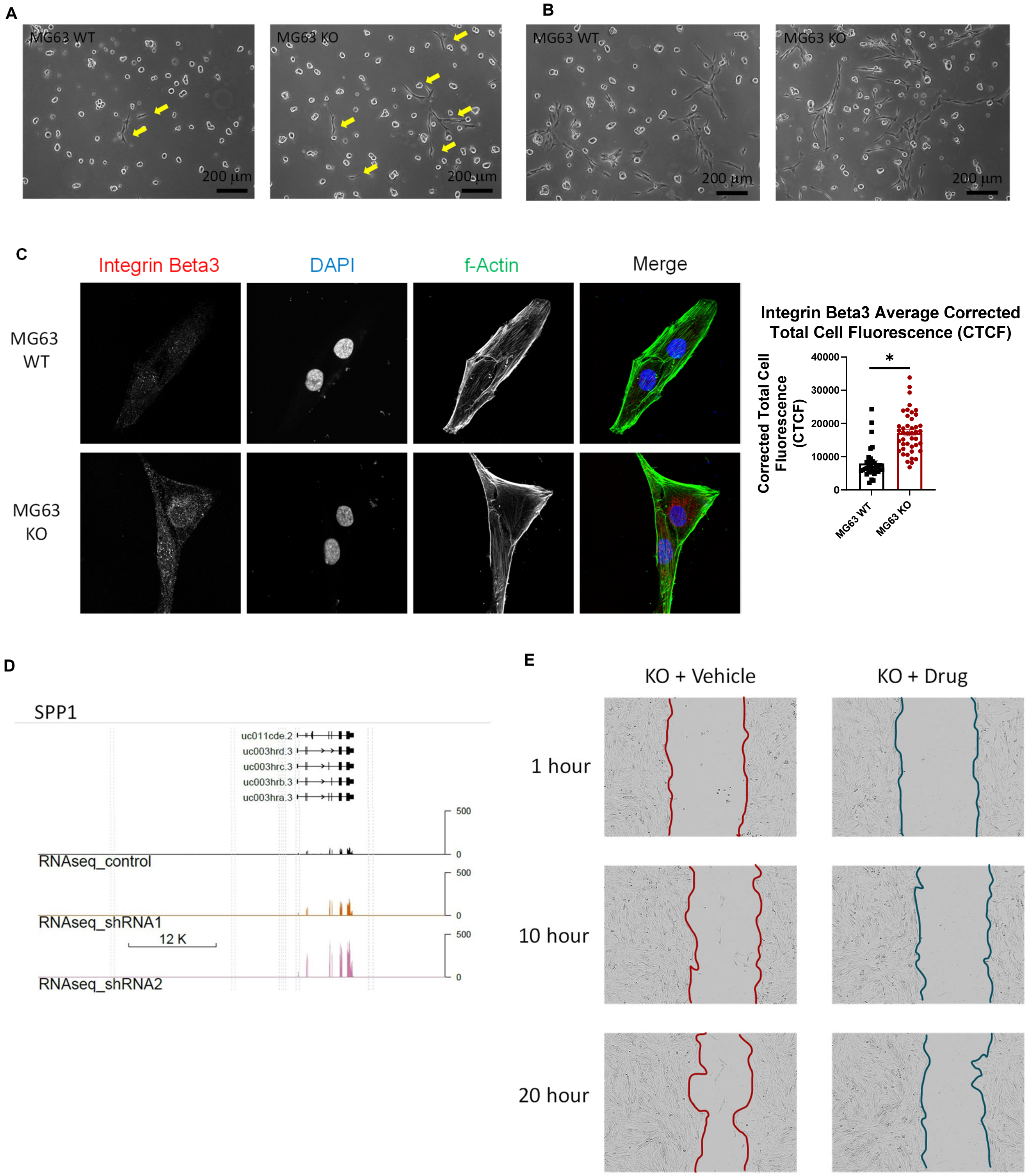

Supplemental Figure 4

A

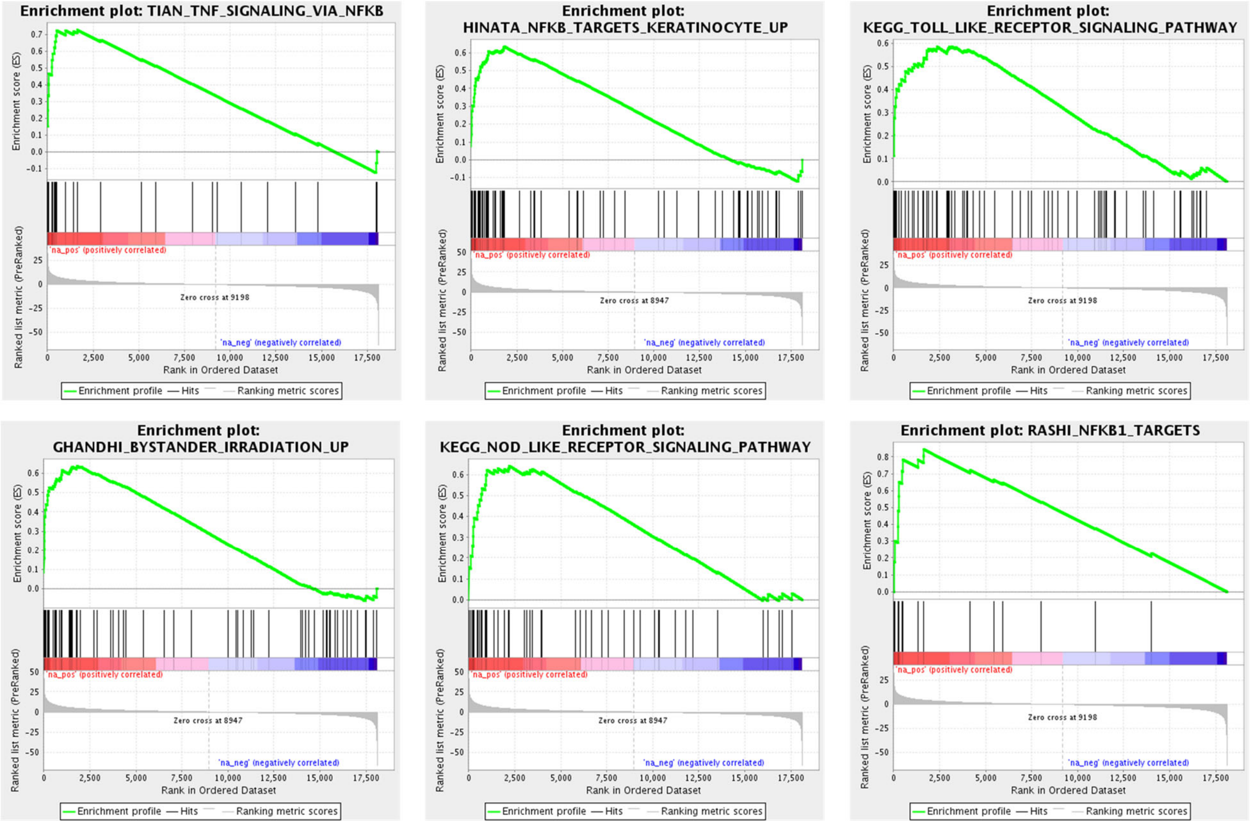

B

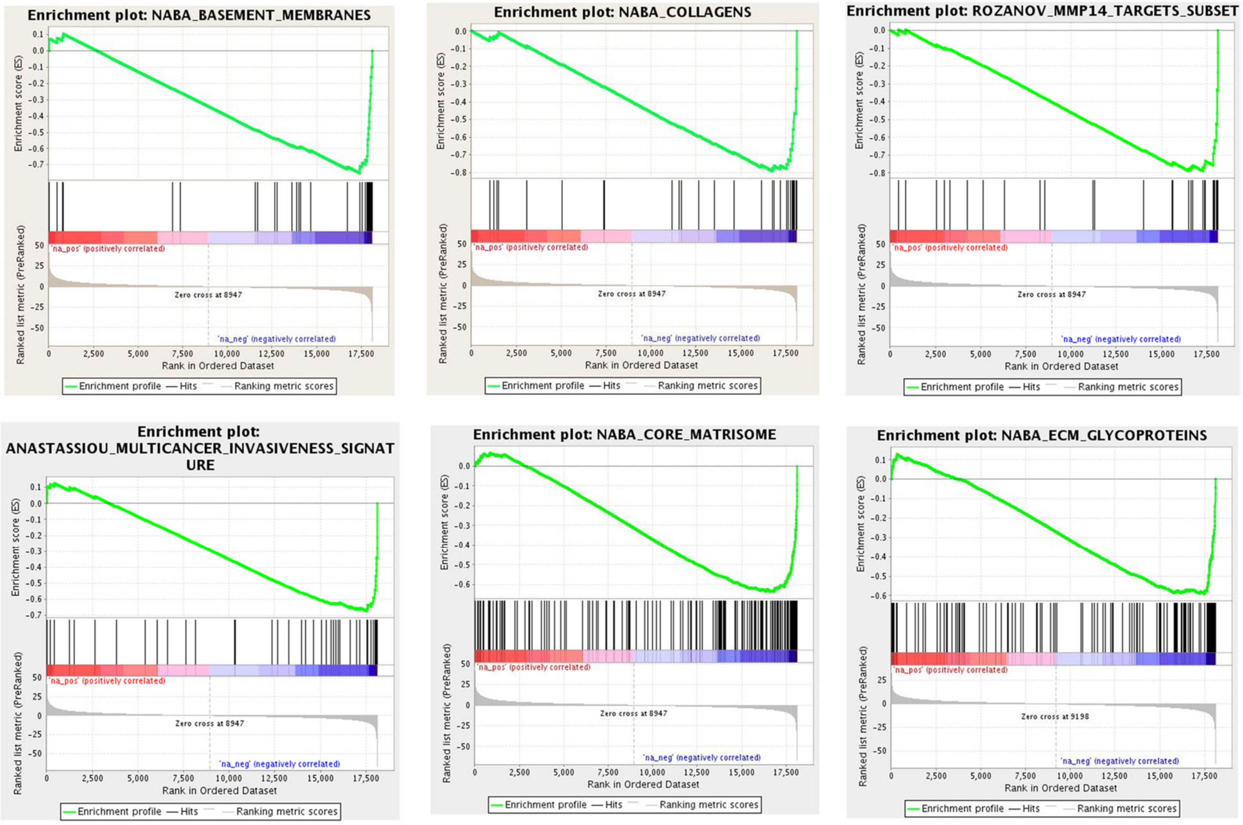

Supplemental Figure 5

A

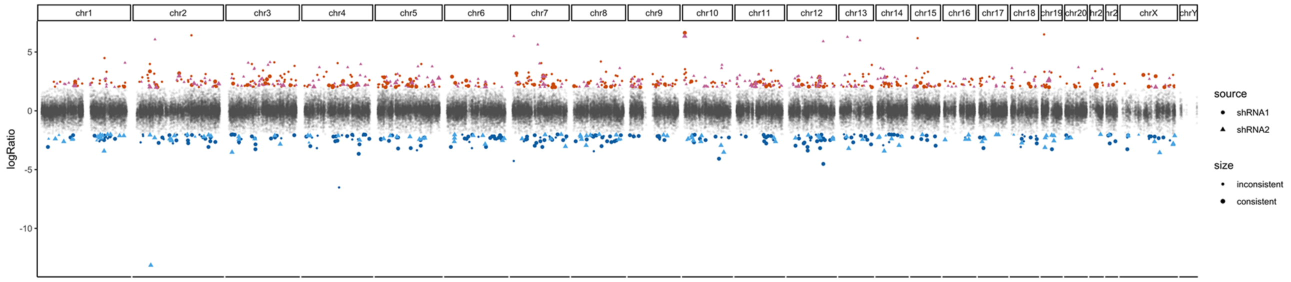

B

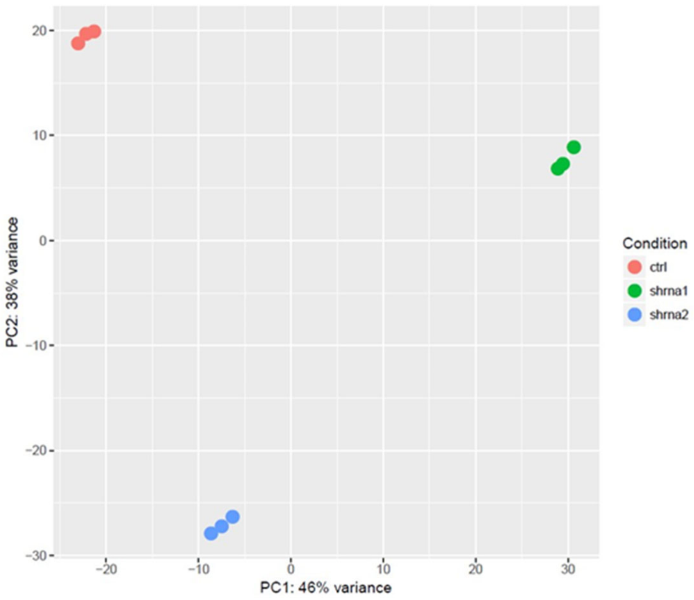

C

Quality control statistics for ATAC-Seq analysis

Reads Total	Reads Aligned	Reads Passing QC	Reads Failed	Duplication Rate	Peaks called	Reads Within Peaks
SD10	125749157	95887755	2883952	0.708002	63761	4834425
SD11	108515306	87226115	3281521	0.508127	60380	6044255
SD12	110613240	86757199	3017028	0.480365	78444	9341844
SD-1	126046234	84366520	1822988	0.742386	83770	5652276
SD-2	104088956	71175083	1534139	0.702606	78714	6404801
SD-3	138801499	92503274	1906453	0.764392	73704	4805285

Supplemental Figure 6

143B WT

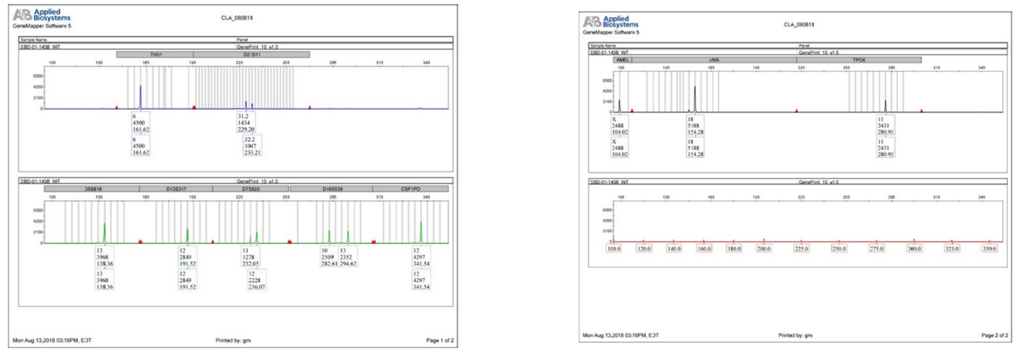

143B KO

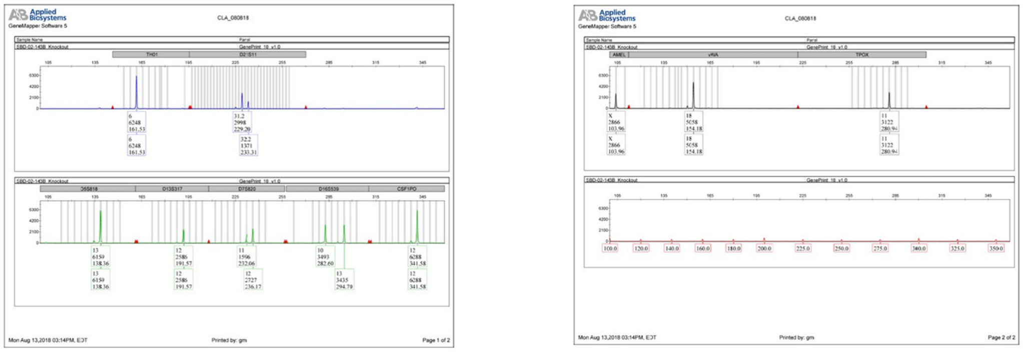

MG63 WT

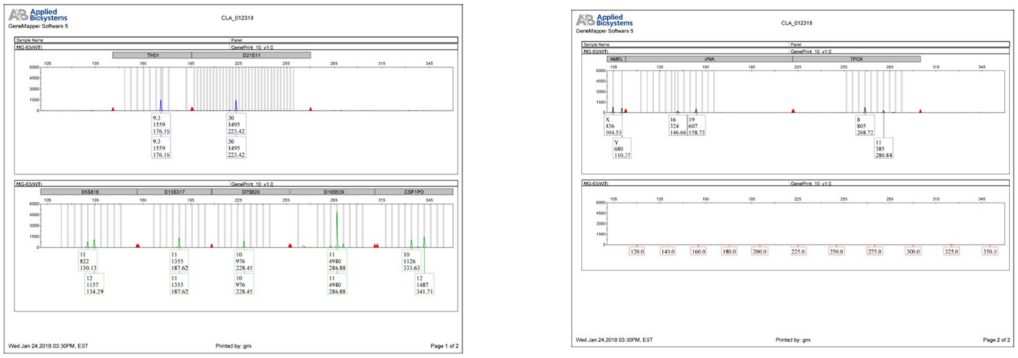

MG63 KO

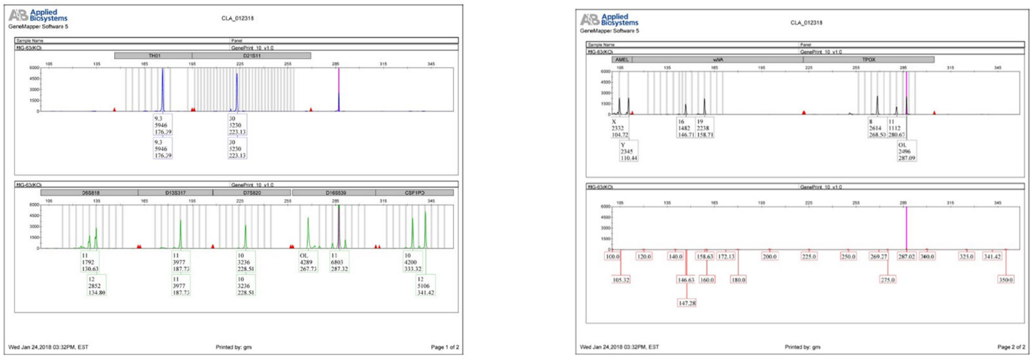

Supplement: Supplemental data [file jciinsight-7-151583-s137.pdf]
